# Epigenetic dysregulation from chromosomal transit in micronuclei

**DOI:** 10.1038/s41586-023-06084-7

**Published:** 2023-06-07

**Authors:** Albert S. Agustinus, Duaa Al-Rawi, Bhargavi Dameracharla, Ramya Raviram, Bailey S. C. L. Jones, Stephanie Stransky, Lorenzo Scipioni, Jens Luebeck, Melody Di Bona, Danguole Norkunaite, Robert M. Myers, Mercedes Duran, Seongmin Choi, Britta Weigelt, Shira Yomtoubian, Andrew McPherson, Eléonore Toufektchan, Kristina Keuper, Paul S. Mischel, Vivek Mittal, Sohrab P. Shah, John Maciejowski, Zuzana Storchova, Enrico Gratton, Peter Ly, Dan Landau, Mathieu F. Bakhoum, Richard P. Koche, Simone Sidoli, Vineet Bafna, Yael David, Samuel F. Bakhoum

**Affiliations:** 1 Human Oncology and Pathogenesis Program, Memorial Sloan Kettering Cancer Center, New York, NY USA; 2 Pharmacology Graduate Program, Weill Cornell Medicine, New York, NY USA; 3 Department of Medicine, Memorial Sloan Kettering Cancer Center, New York, NY USA; 4 Department of Computer Science, University of California, San Diego, La Jolla, CA USA; 5 New York Genome Center, New York, NY USA; 6 Department of Ophthalmology and Visual Science, Yale University School of Medicine, New Haven, CT USA; 7 Department of Biochemistry, Albert Einstein College of Medicine, New York, NY USA; 8 School of Engineering, University of California, Irvine, Irvine, CA USA; 9 Tri-institutional MD-PhD Program, New York, NY USA; 10 Computational Oncology, Department of Epidemiology and Biostatistics, Memorial Sloan Kettering Cancer Center, New York, NY USA; 11 Department of Pathology and Laboratory Medicine, Memorial Sloan Kettering Cancer Center, New York, NY USA; 12 Department of Cell and Developmental Biology, Weill Cornell Medicine, New York, NY USA; 13 Molecular Biology Program, Memorial Sloan Kettering Cancer Center, New York, NY USA; 14 Department of Molecular Genetics, University of Kaiserslautern, Kaiserslautern, Germany; 15 Department of Pathology, School of Medicine, Stanford University, Stanford, CA USA; 16 Department of Pathology, University of Texas Southwestern Medical Center, Dallas, TX USA; 17 Meyer Cancer Center, Weill Cornell Medicine, New York, NY USA; 18 Department of Pathology, Yale University School of Medicine, New Haven, CT USA; 19 Yale Cancer Center, Yale University, New Haven, CT USA; 20 Center for Epigenetics Research, Memorial Sloan Kettering Cancer Center, New York, NY USA; 21 Chemical Biology Program, Memorial Sloan Kettering Cancer Center, New York, NY USA; 22 Tri-institutional PhD Program in Chemical Biology, New York, NY USA; 23 Department of Radiation Oncology, Memorial Sloan Kettering Cancer Center, New York, NY USA

**Keywords:** Histone post-translational modifications, Tumour heterogeneity, Chromosomes

## Abstract

Chromosomal instability (CIN) and epigenetic alterations are characteristics of advanced and metastatic cancers^1–4^, but whether they are mechanistically linked is unknown. Here we show that missegregation of mitotic chromosomes, their sequestration in micronuclei^5,6^ and subsequent rupture of the micronuclear envelope^7^ profoundly disrupt normal histone post-translational modifications (PTMs), a phenomenon conserved across humans and mice, as well as in cancer and non-transformed cells. Some of the changes in histone PTMs occur because of the rupture of the micronuclear envelope, whereas others are inherited from mitotic abnormalities before the micronucleus is formed. Using orthogonal approaches, we demonstrate that micronuclei exhibit extensive differences in chromatin accessibility, with a strong positional bias between promoters and distal or intergenic regions, in line with observed redistributions of histone PTMs. Inducing CIN causes widespread epigenetic dysregulation, and chromosomes that transit in micronuclei experience heritable abnormalities in their accessibility long after they have been reincorporated into the primary nucleus. Thus, as well as altering genomic copy number, CIN promotes epigenetic reprogramming and heterogeneity in cancer.

## Abstract (web summary)

Missegregated chromosomes that are sequestrated in micronuclei are subject to changes in histone modifications leading to abnormalities in chromatin accessibility that remain long after the chromosomes have been reincorporated into the primary nucleus.

## Main

CIN drives tumour progression, in part through the generation of genomic copy-number heterogeneity, which serves as a substrate for natural selection^2,4,8–10^. CIN is associated with metastasis^11^, therapeutic resistance^12^ and immune evasion^13,14^, and it results from the missegregation of chromosomes during mitosis^15^. A hallmark of cancer cells with CIN is the presence of lagging chromosomes in anaphase^15^. Missegregated chromosomes frequently end up in micronuclei, which have envelopes that are rupture-prone, exposing their genomic content to the cytosol^6,11,16,17^. Widespread DNA damage from such rupture can catalyse genomic abnormalities, including complex chromosomal rearrangements known as chromothripsis^18,19^. Chromosomes that are encapsulated in micronuclei are often wholly or partly re-integrated into the primary nucleus after mitosis, and as such can propagate genetic abnormalities acquired, while in the micronucleus, to daughter cells^18,19^. In contrast to the genomic ramifications of chromosome missegregation, little is known about the effects of chromosomal transit in micronuclei on the integrity of the epigenetic landscape.

## Altered histone PTMs in micronuclei

**Fig. 1:**
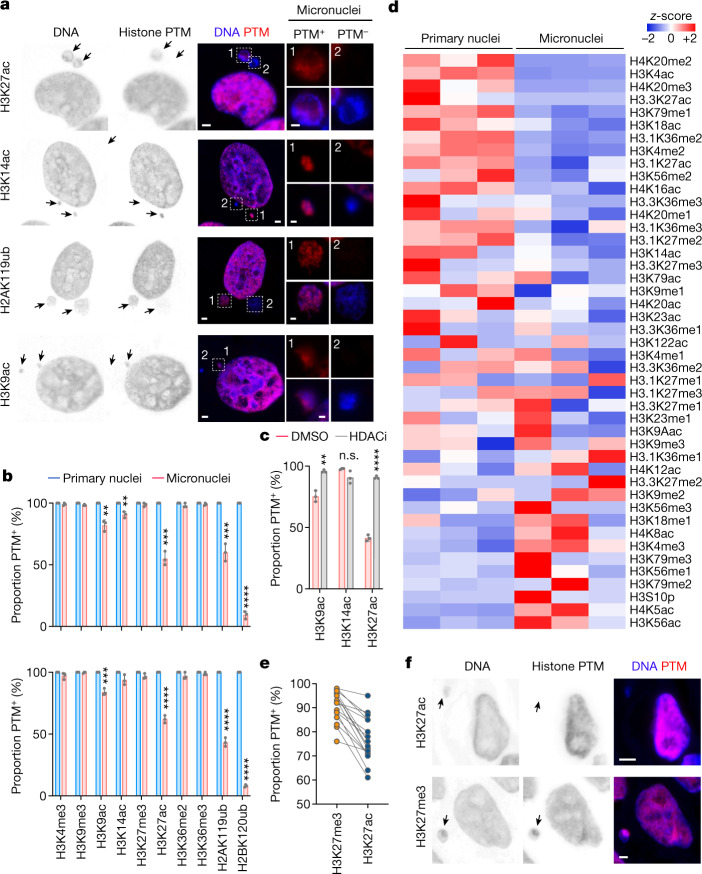
Distinct histone PTMs in micronuclei. **a**, Representative immunofluorescence images from three biological replicates of micronucleated MDA-MB-231 cells stained for DNA (blue) and histone post-translational modifications (PTMs; red). White outlined boxes show a magnified view of micronuclei. **b**, Percentage of primary nuclei (blue) and micronuclei with particular histone PTMs (red) from an immunofluorescence experiment using human MDA-MB-231 (top) and mouse 4T1 (bottom) cells. **P *<* 0.01, ***P < 0.001, ****P *<* 0.0001, two-sided *t-*test, *n* = 3 biological replicates; data represent mean ± s.d.; n.s., not significant. **c**, Percentage of micronuclei with particular histone PTMs from an immunofluorescence experiment in MDA-MB-231 cells treated with DMSO vehicle control or vorinostat (HDAC inhibitor, HDACi). Statistical details are the same as in **b**. **d**, Heat map of *z*-scores of the relative abundance of histone PTMs from histone mass spectrometry in isolated micronuclei and primary nuclei of mouse 4T1 cells; *n* = 3. **e**, Percentage of micronuclei with H3K27me3 and H3K27ac from immunofluorescence staining in human high-grade serous ovarian cancer (HGSOC) tumour samples; *n* = 16, statistical significance tested using two-sided paired *t*-test; *p* < 0.0001. **f**, Representative immunofluorescence images of 16 human HGSOC tumour samples stained for DNA (blue) and either H3K27me3 or H3K27ac (red). In **a** and **f**, arrows, micronuclei; scale bars, 2 µm. Source data

**Extended Data Fig. 1:**
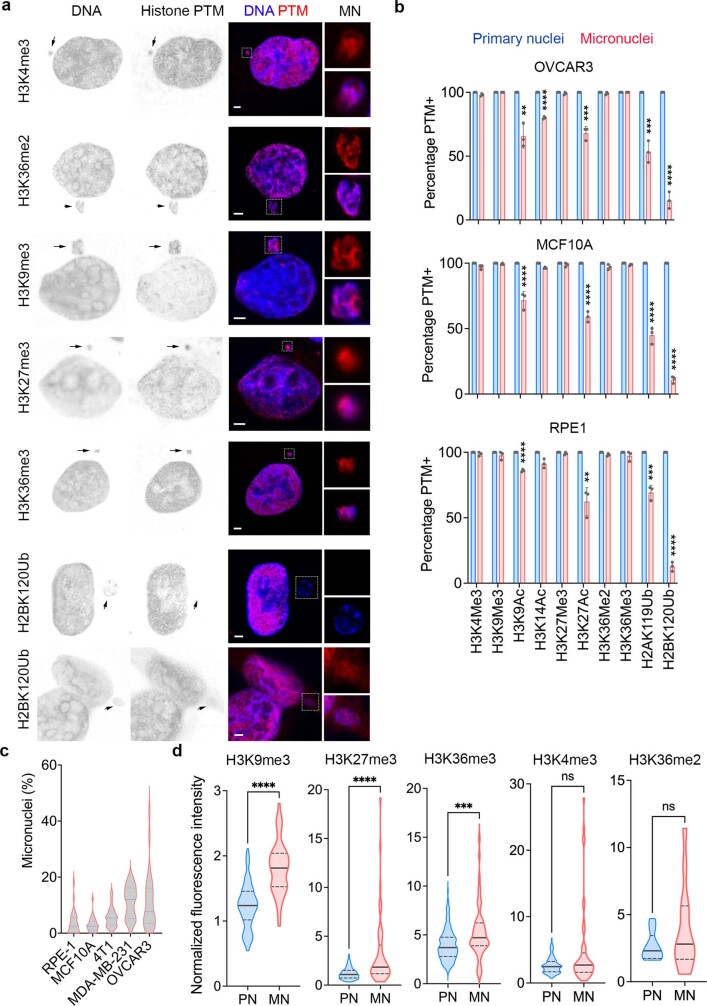
Micronuclei have distinct histone PTM profiles compared with primary nuclei. **a**, Representative immunofluorescence images from 3 biological replicates of micronucleated MDA-MB-231 cells stained for DNA (blue) and histone posttranslational modifications (PTMs) (red), arrows point to micronuclei, scale bars 2 µm. Insets show magnified view of micronuclei (white outlined box). **b**, Percentage of primary nuclei and micronuclei with given histone PTMs from immunofluorescence experiment in OVCAR3, MCF10A, and RPE-1 cells, ** *p* < 0.01, *** *p* < 0.001, **** *p* < 0.0001, two-sided *t-*test, n = 3 biological replicates, bars represent mean ± SD. **c**, Violin plots showing the distribution of micronuclei percentage to primary nuclei in various cell lines used in the immunofluorescence quantification experiments. Values were obtained from 3 biological replicates. In each biological replicate, 10 fields of view under 63x magnification were observed. Solid and dashed lines in the plot represent the median and quartiles, respectively. **d**, Violin plots showing the normalized immunofluorescence intensity distribution of histone PTMs in: 55 micronuclei (MN) and 104 accompanying primary nuclei (PN) for H3K9Me3, 52 MN and 213 accompanying PN for H3K27Me3, 56 MN and 162 accompanying PN for H3K36Me3, 52 MN and 213 accompanying PN for H3K4Me3, 13MN and accompanying PN for H3K36Me2. Experiments were done on MDA-MB-231 cells; ns not significant, *** *p* < 0.001, **** *p* < 0.0001, two-sided Mann-Whitney test. Solid and dashed lines in the plot represent the median and quartiles, respectively. Source data

**Extended Data Fig. 2:**
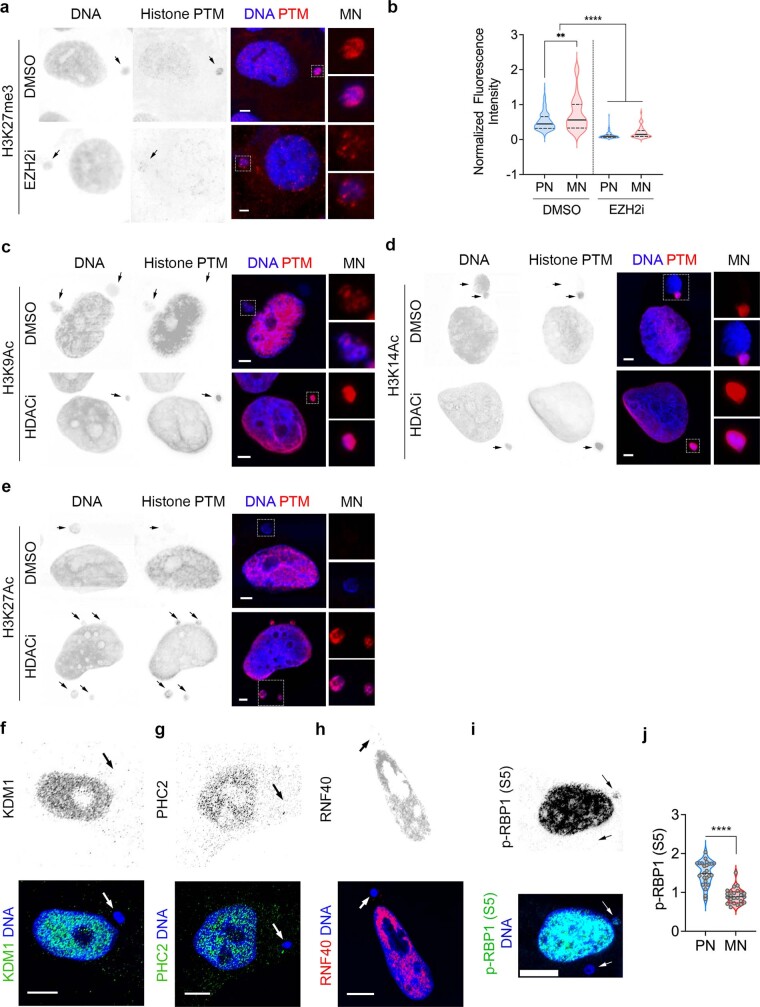
Epigenetic modifying drugs and epigenetic enzymes alter histone PTMs in micronuclei. **a**, Representative immunofluorescence images from 3 biological replicates of micronucleated MDA-MB-231 cells stained for DNA (blue) and histone PTMs (red) treated with vehicle control (DMSO) or GSK126 EZH2 inhibitor (EZH2i), arrows point to micronuclei, scale bars 2 µm. Insets show magnified view of micronuclei (white outlined box). **b**, Violin plot showing normalized immunofluorescence intensity distribution of H3K27Me3 from immunofluorescence experiment in MDA-MB-231 cells. Cells were either treated with vehicle control (DMSO) or GSK126 EZH2 inhibitor (EZH2i); n = 52 micronuclei (MN) and 222 accompanying primary nuclei (PN) for DMSO-treated cells; n = 58 MN and n = 196 accompanying PN for EZH2i-treated cells; ** *p* < 0.01, **** *p* < 0.0001, two-sided Mann-Whitney test. Solid and dashed lines in the plot represent the median and quartiles, respectively. **c**–**e**, Representative immunofluorescence images from 3 biological replicates of micronucleated MDA-MB-231 cells stained for DNA (blue) and histone PTMs (red) treated with vehicle control (DMSO) or vorinostat HDAC Inhibitor (HDACi). Arrows point to micronuclei, scale bars 2 µm. Insets show magnified view of micronuclei (white outlined box). **f**–**h**, Representative immunofluorescence image from 3 biological replicates of micronucleated MDA-MB-231 cells stained for DNA (blue) and various epigenetic enzymes/complex members; **f**: KDM1 (green), **g**: PHC2 (green), **h**: RNF40 (red). Arrows point to micronuclei. Scale bars 2 µm. **i**, Representative immunofluorescence image from 3 biological replicates of micronucleated MDA-MB-231 cells stained for DNA in blue and phospho-RPB1 carboxy-terminal domain (serine 5), a functionally active RNA polymerase II (RNAPII) subunit, in green. Arrows point to micronuclei. Scale bars 2 µm. **j**, Violin plots showing normalized immunofluorescence intensity distribution of phospho-RPB1 carboxy-terminal domain (serine 5) from immunofluorescence experiment in MDA-MB-231, n = 25 MN and PN, **** *p* < 0.0001, two-sided Mann-Whitney test. Solid and dashed lines in the plot represent the median and quartiles, respectively. Source data

To determine the epigenetic consequences of chromosome sequestration in micronuclei, we used immunofluorescence microscopy to assess the status of canonical histone PTMs in human non-transformed mammary epithelial cells (MCF10A), telomerase-immortalized retinal pigment epithelial cells (RPE-1), high-grade serous ovarian cancer (HGSOC) cells (OVCAR-3) and both human and mouse triple-negative breast-cancer cells (MDA-MB-231 and 4T1, respectively). We observed substantial differences in the distribution of histone PTMs when comparing primary nuclei and micronuclei. Micronuclei had reductions in the transcriptional activating mark, lysine acetylation, at multiple residues along the histone H3 tail (H3K9ac, H3K27ac and, to a lesser extent, H3K14ac, which was lost only in tumour cells). Moreover, the two canonical ubiquitination sites on histones, the repressive mono-ubiquitination of histone H2A (H2AK119ub) and the gene-body-specific mono-ubiquitination of histone H2B (H2BK120ub), exhibited substantial reductions, with an almost complete loss of H2BK120ub (Fig. 1a,b and Extended Data Fig. 1a,b). Changes to histone PTM patterns in micronuclei were remarkably conserved among non-transformed and cancer-derived cells, as well as across species, regardless of the basal rate of micronucleus formation (Fig. 1b and Extended Data Fig. 1b,c). Although some important lysine methylation events on histone H3 were preserved in micronuclei, others, such as H3K9me3, H3K27me3 and H3K36me3, were enriched (Extended Data Fig. 1d). Treatment with the pan-histone deacetylase (HDAC) inhibitor vorinostat led to near-complete restoration of H3K9ac, H3K14ac and H3K27ac signals in micronuclei, whereas treatment with the EZH2 inhibitor GSK126 led to a reduction in H3K27me3 staining intensity (Fig. 1c and Extended Data Fig. 2a–e). These changes in histone PTMs indicate an altered balance of histone-modifying enzymes, many of which were found to be absent in micronuclei (Extended Data Fig. 2f–h). Furthermore, we observed reduced phosphorylation of RNA polymerase II subunit B1 (RPB1) in micronuclei compared with primary nuclei, suggesting reduced transcriptional activity (Extended Data Fig. 2i,j).

**Extended Data Fig. 3:**
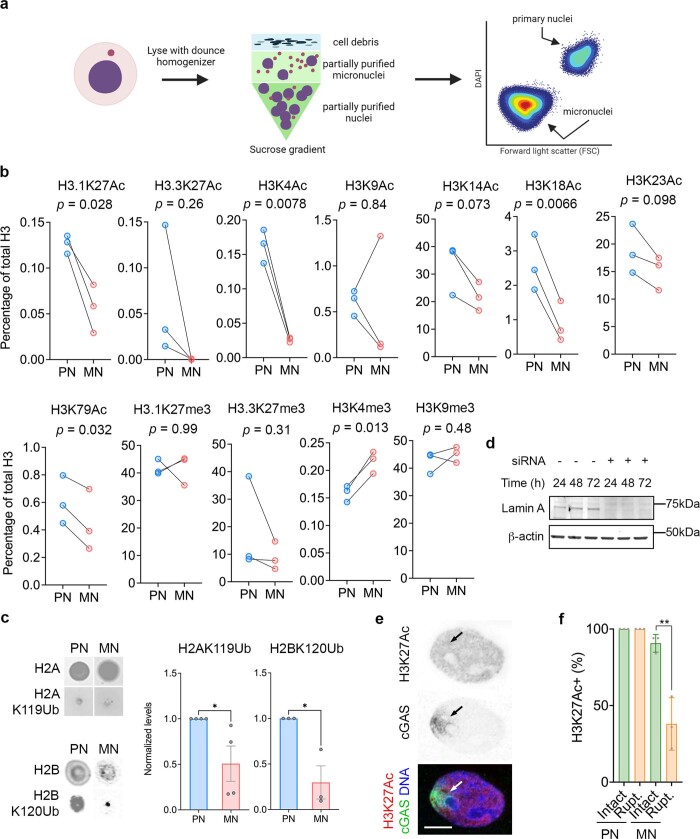
Mass spectrometry analysis of histone PTMs and epigenetic enzymes profiling in primary nuclei and micronuclei. **a**, Micronuclei purification experiment schematic using cell lysis followed by sucrose gradient ultracentrifugation and FACS (figure created on Biorender.com). **b**, Abundance of various histone H3 PTMs relative to total histone H3 levels obtained from bottom-up histone mass spectrometry performed on isolated micronuclei (MN) and primary nuclei (PN) of 4T1 cells, n = 3 biological replicates, statistical significance tested using two-sided paired t-test, no adjustments made for multiple comparisons. **c**, (Left) Dot blots of H2AK119Ub and H2BK120Ub as well as total histones H2A and H2B as control from isolated micronuclei (MN) and primary nuclei (PN) of MDA-MB-231 cells. (Right) Relative quantification of H2AK119Ub and H2BK120Ub level normalized to total histones H2A and H2B, respectively, * *p* < 0.05, two-sided *t-*test, n = 4 biological replicates (H2AK119Ub), n = 3 biological replicates (H2BK120Ub), bars represent mean ± SD. **d**, Representative immunoblotting result from 3 biological replicates of lamin A in MDA-MB-231 cells treated with scrambled siRNA control (−) and siRNA for lamin A (+) for 24, 48, and 72 h with β-actin as loading control. **e**, Representative image from 3 biological replicates of MDA-MB-231 cells treated with shRNA for lamin A, stained for DNA (blue), H3K27ac (red), and cGAS (green). Arrows point to cGAS-positive area which indicates lamin rupture. Scale bar 5 µm. **f**, Percentage of intact (green) and ruptured (rupt.; gold) primary nuclei (PN) or micronuclei (MN) that are positive for H3K27ac immunofluorescence staining from MDA-MB-231 cells, ** *p* < 0.01, two-sided *t-*test, n = 3 biological replicates, bars represent mean ± SD. Source data

Next we purified and separated primary nuclei from micronuclei of MDA-MB-231 and 4T1 cells, as previously described^20^ (Methods and Extended Data Fig. 3a). A quantitative mass-spectrometry survey of canonical histone PTMs in micronuclei confirmed reductions in acetylation on multiple lysine residues on histones H3 and H4, when compared with primary nuclei (Fig. 1d and Extended Data Fig. 3b). Overall, the distribution and levels of histone marks were markedly distinct between primary nuclei and micronuclei (Fig. 1d). Immunoblot analysis confirmed the loss of both mono-ubiquitination marks (H2AK119ub and H2BK120ub) in micronuclei (Extended Data Fig. 3c). In line with our findings in cell lines, there was relative preservation of H3K27me3 compared with H3K27ac in micronuclei in 16 human HGSOC samples (Fig. 1e,f).

## Micronuclear defects disrupt histone PTM

**Fig. 2:**
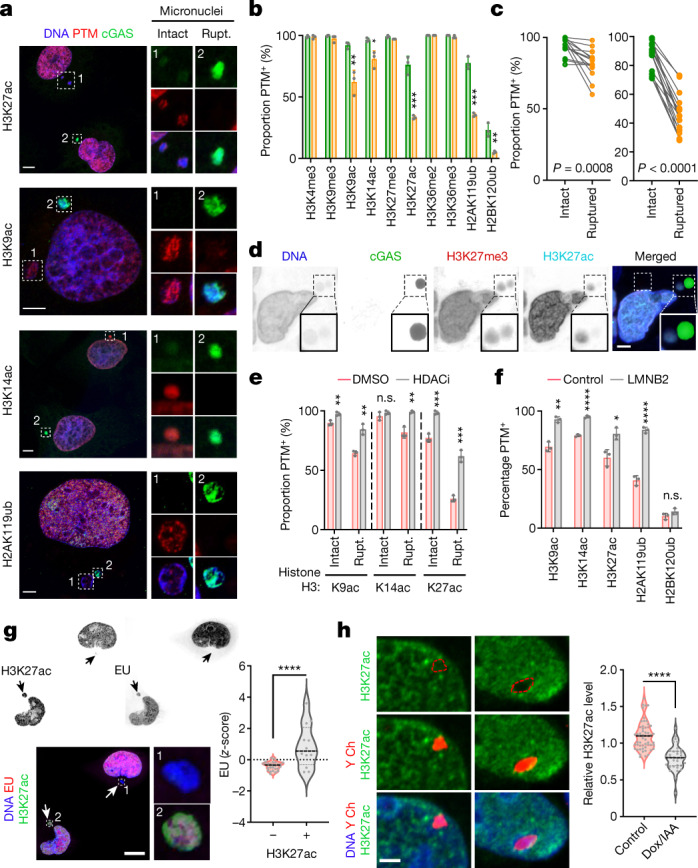
Micronuclear rupture and chromosome missegregation alter histone post-translational modifications in micronuclei. **a**, Representative images of MDA-MB-231 cells stained for DNA (blue), histone PTMs (red) and cGAS (green). White outlined boxes show a magnified view of micronuclei, either intact or ruptured (Rupt.). **b**, Percentage of intact (green bars) and ruptured (yellow) micronuclei with histone PTMs in MDA-MB-231 cells; *P < 0.05, **P < 0.01, ***P < 0.001, two-sided *t-*test, *n* = 3; bars show mean ± s.d. **c**, Percentage of intact and ruptured micronuclei with H3K27me3 (left, *P* = 0.0008) and H3K27ac (right, *P* < 0.0001) in human high-grade serous ovarian cancer (HGSOC) samples; *n* = 16, two-sided paired *t-*test. **d**, Representative images from 16 human HGSOC samples stained for DNA (blue), cGAS (green), H3K27me3 (red) and H3K27ac (cyan). **e**, Percentage of intact and ruptured micronuclei with histone PTMs in DMSO-treated (red bars) or vorinostat-treated (grey) MDA-MB-231 cells; **P < 0.01, ***P < 0.001, two-sided *t-*test, *n* = 3; data represent mean ± s.d. **f**, Percentage of micronuclei with histone PTMs in control (red bars) and lamin-B2-expressing (LMNB2; grey) MDA-MB-231 cells; *P < 0.05, **P < 0.01, ****P < 0.0001, two-sided *t-*test, *n* = 3; data represent mean ± s.d. **g**, Representative images of MDA-MB-231 cells with micronuclei stained for DNA (blue), H3K27ac (green) and nascent RNA (red). Violin plots show the *z*-score of ethynyluridine (EU) intensity; ****P < 0.0001, two-sided Mann–Whitney *U*-test, *n* = 3. Solid and dotted bars represent the median and quartiles, respectively. **h**, Representative fluorescence in situ hybridization (FISH) images of CEN-SELECT DLD-1 cells treated with DMSO (control, left) or Dox/IAA (right) stained for Y chromosome (red), DNA (blue) and H3K27ac (green). Violin plots show the H3K27ac immunofluorescence intensity of DLD-1 cells treated with either DMSO (control) or Dox/IAA; ****P < 0.0001, two-sided Kolmogorov-Smirnov test, *n* = 3. Scale bars, 2 µm (**d**), 5 µm (**a** and **h**) and 10 µm (**g**). Source data

**Extended Data Fig. 4:**
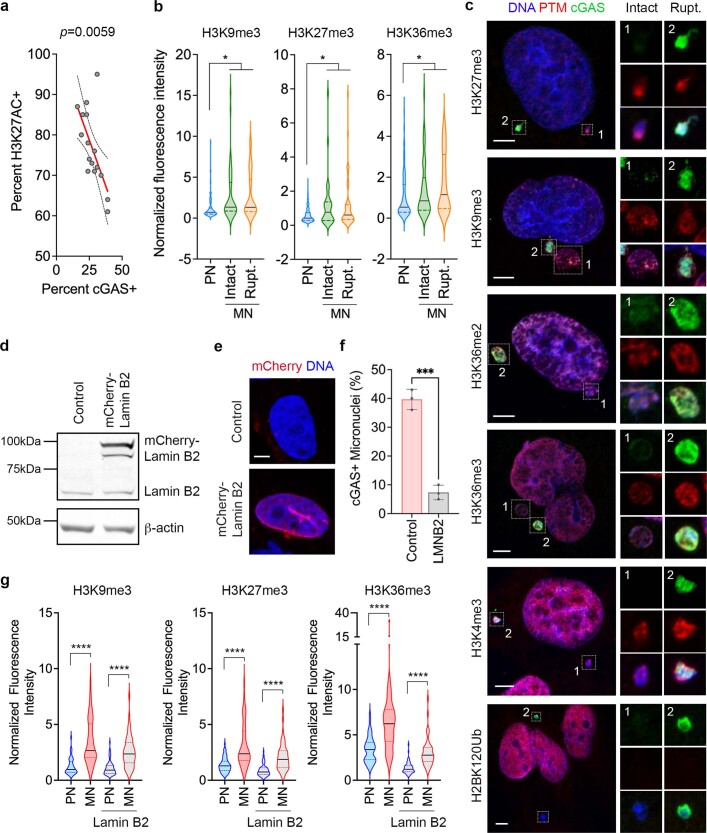
Impact of micronuclear rupture on histone PTMs. **a**, Sample-level correlation between percentages of micronuclei with H3K27ac and cGAS staining in human high grade serous ovarian cancer (HGSOC) tumor samples (n = 16), 100 micronuclei counted/sample. statistical significance tested using two-sided *t-*test, no adjustments made for multiple comparisons. **b**, Violin plot showing normalized immunofluorescence intensity of histone PTMs in MDA-MB-231 cells’ PN, intact MN or ruptured (rupt.) MN; * *p* < 0.05, two-sided Mann-Whitney test). For H3K9Me3: n = 209 PN, 45 intact MN, and 51 ruptured MN; for H3K27Me3: n = 388 PN, 36 intact MN, and 53 ruptured MN; for H3K36Me3: n = 213 PN, 54 intact MN, and 39 ruptured MN. Solid and dashed lines in the plot represent the median and quartiles, respectively. **c**, Representative immunofluorescence images from 3 biological replicates of micronucleated MDA-MB-231 cells stained for DNA (blue), histone PTMs (red), and cGAS (green), scale bars 10 µm. Insets show magnified view of micronuclei (white outlined box). **d**, Representative immunoblotting results from 3 biological replicates of lamin B2 in control and mCherry-lamin B2 overexpressing MDA-MB-231 cells with β-actin as loading control. **e**, Representative immunofluorescence image of control and mCherry-lamin B2 overexpressing MDA-MB-231 cells stained with DNA (blue), scale bar 10 µm. **f**, Percentage of micronuclei with cGAS positive immunofluorescence staining in control and mCherry-lamin B2 overexpressing MDA-MB-231 cells (LMNB2); *** *p* < 0.001, two-sided *t-*test, n = 3 biological replicates, bars represent mean ± SD. **g**, Violin plot showing normalized immunofluorescence intensity of histone PTMs in primary nuclei (PN) and micronuclei (MN) of control and mCherry-lamin B2 (lamin B2) overexpressing MDA-MB-231 cells. For H3K9Me3: n = 99 PN (WT), 53 MN (WT), 73 PN (lamin B2), and 55 MN (lamin B2); for H3K27Me3: n = 73 PN (WT), 50 MN (WT), 82 PN (lamin B2), and 56 MN (amin B2); for H3K36Me3: n = 50 PN (WT), 50 MN (WT), 68 PN (lamin B2), and 50 MN (lamin B2); **** *p* < 0.0001, two-sided Mann-Whitney test, solid and dashed lines in the plot represent the median and quartiles, respectively. Source data

To test whether abnormalities in histone PTMs result from rupture of the micronuclear envelope^6,7^ we co-stained histone PTMs with cGAS, a cytosolic double-stranded DNA sensor^11,16,17,21^. Micronuclei lacking histone H3 acetylation (K9ac, K14ac and K27ac) and H2AK119ub were more likely to display cGAS colocalization (Fig. 2a,b). This phenomenon was restricted to micronuclei because inducing primary-nuclear rupture by depleting lamin A did not alter H3K27ac staining intensity (Extended Data Fig. 3d–f). In human HGSOC there was widespread loss of H3K27ac in micronuclei with cGAS staining, in contrast to slight reductions in H3K27me3 (Fig. 2c,d and Extended Data Fig. 4a), which we attribute to reduced sensitivity in detecting histone PTMs in preserved human tumour samples compared with cell lines (Supplementary Fig. 2). Treatment of MDA-MB-231 cells with vorinostat restored histone H3 lysine acetylation in all micronuclei (Fig. 2e).

Notably, H2BK120ub was lost in nearly all micronuclei, whereas many stable heterochromatin-associated marks (H3K9me3, H3K27me3 and H3K36me3) were enriched, irrespective of micronuclear-rupture status (Fig. 2b and Extended Data Fig. 4b,c). To test the contribution of such rupture to changes in histone PTMs, we expressed lamin B2, which suppresses micronuclear-envelope collapse^7^, as demonstrated by a reduction in the fraction of micronuclei with cGAS staining (Extended Data Fig. 4d–f). Lamin B2 expression selectively rescued abnormalities in rupture-associated histone PTMs (H3K9ac, H3K14ac, H3K27ac and H2AK119ub) with no impact on changes unrelated to rupture, such as loss of H2BK120ub or enrichment of lysine trimethyl marks on histone H3 (Fig. 2f and Extended Data Fig. 4g).

**Extended Data Fig. 5:**
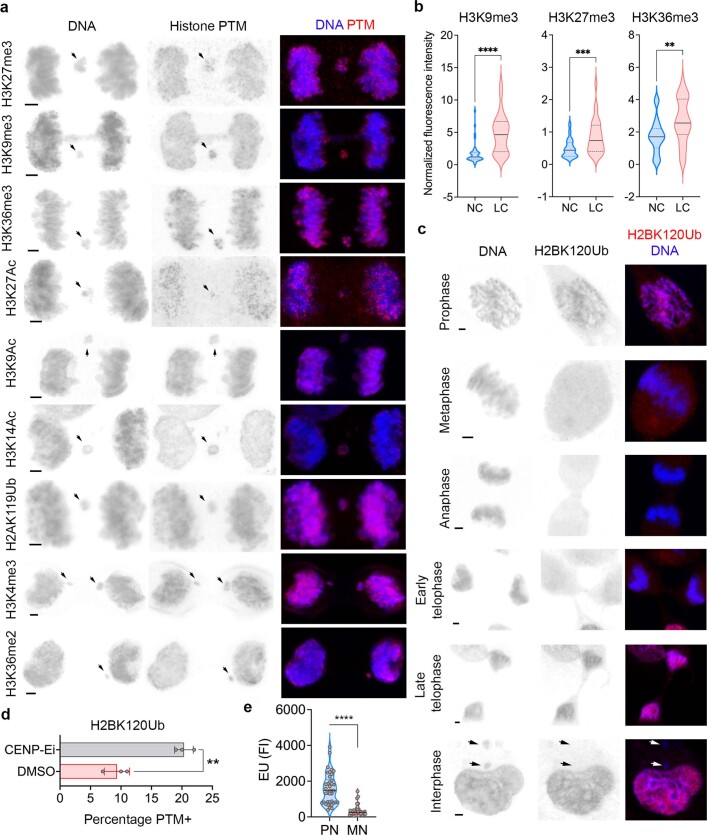
Mitotic errors are associated with abnormalities in histone PTMs. **a**, Representative immunofluorescence image of MDA-MB-231 cells undergoing anaphase with lagging chromosome (pointed by arrows) stained for DNA (blue) and histone PTMs (red). Scale bars 2 µm. **b**, Violin plots showing the normalized immunofluorescence intensity distribution of histone PTMs on normal chromosomes (NC) and lagging chromosomes (LC) of MDA-MB-231 cells during mitosis. For H3K9Me3: n = 40 NCs and LCs, for H3K27Me3: n = 42 NCs and 28 LCs, for H3K36Me3: n = 40 NCs and 31 LCs; ** *p* < 0.01, *** *p* < 0.001, **** *p* < 0.0001, two-sided Mann-Whitney test, solid and dashed bars in the plot represent the median and quartiles, respectively. **c**, Representative immunofluorescence images of MDA-MB-231 cells at different stages of the cell cycle, stained for DNA (blue) and H2BK120Ub (red), arrows point towards micronuclei, scale bars 2 µm. **d**, Percentage of H2BK120Ub-positive micronuclei immunofluorescence staining on vehicle (DMSO)-treated or CENP-E inhibitor (CENP-Ei, GSK923295)-treated MDA-MB-231 cells; ** *p* < 0.01, two-sided *t-test*, n = 3 biological triplicates, bars represent mean ± SD. **e**, Violin plot showing the fluorescence intensity quantification from EU staining experiment in MN and PN of MDA-MB-231 cells; n = 38 MN and PN, **** *p* < 0.0001, two-sided Mann-Whitney test. Solid and dashed bars in the plot represent the median and quartiles, respectively. Source data

We investigated whether rupture-unrelated changes in histone PTMs could arise during the previous round of mitosis and are subsequently inherited with the lagging chromosome, thereby pre-dating the formation of the micronucleus. We examined the fluorescence intensity of 10 canonical histone PTMs during anaphase, comparing lagging chromosomes with the remaining normally segregating chromosomes. H3K9me3, H3k27me3 and H3K36me3, which were all enriched in micronuclei, were also enriched in lagging chromosomes compared with their normally segregating counterparts (Extended Data Fig. 5a,b).

H2BK120ub was conspicuously absent from mitotic cells, as previously reported^22^, from prometaphase to early telophase (Extended Data Fig. 5c). Whereas chromosomes in the primary nucleus quickly regained this mark in late telophase, micronuclei did not (Extended Data Fig. 5c). We reasoned that this unequal redeposition was due to biased subcellular localization of the H2BK120 ubiquitin ligase complex (RNF20/40) near the spindle poles^23^, thus placing lagging chromosomes out of reach. We induced the formation of micronuclei arising from chromosomes that missegregate while remaining near the spindle poles, without transiting through the midzone, by inhibiting CENP-E, which is a kinesin-like motor protein that promotes chromosome congression to the metaphase plate^24^. Inhibiting CENP-E with GSK923295^25^ led to a significant increase in micronuclei containing H2BK120ub (Extended Data Fig. 5d).

Next, we pulse-treated MDA-MB-231 cells with fluorescently labelled 5-ethynyluridine and assessed its incorporation in primary nuclei and micronuclei. As expected, there was an overall reduction in transcriptional activity in micronuclei relative to primary nuclei; however, we observed significantly increased 5-ethynyluridine incorporation in micronuclei that have retained H3K27ac compared with those that have lost this PTM (Fig. 2g and Extended Data Fig. 5e). We examined whether changes in histone PTMs can persist during the subsequent cell cycle, once the chromosome that was previously in a micronucleus is reintegrated into the primary nucleus. We used an inducible Y-chromosome-specific missegregation system established in otherwise chromosomally stable DLD-1 colorectal cancer cells^18,26^, staining for H3K27ac. This approach allows efficient doxycycline and auxin (DOX/IAA)-dependent micronucleation of the Y chromosome without affecting the autosomes or the X chromosome^26^. H3K27ac associated with the Y chromosome was significantly reduced in cells treated for 24 h with DOX/IAA compared with their untreated counterparts (Fig. 2h).

## Altered chromatin accessibility in micronuclei

**Fig. 3:**
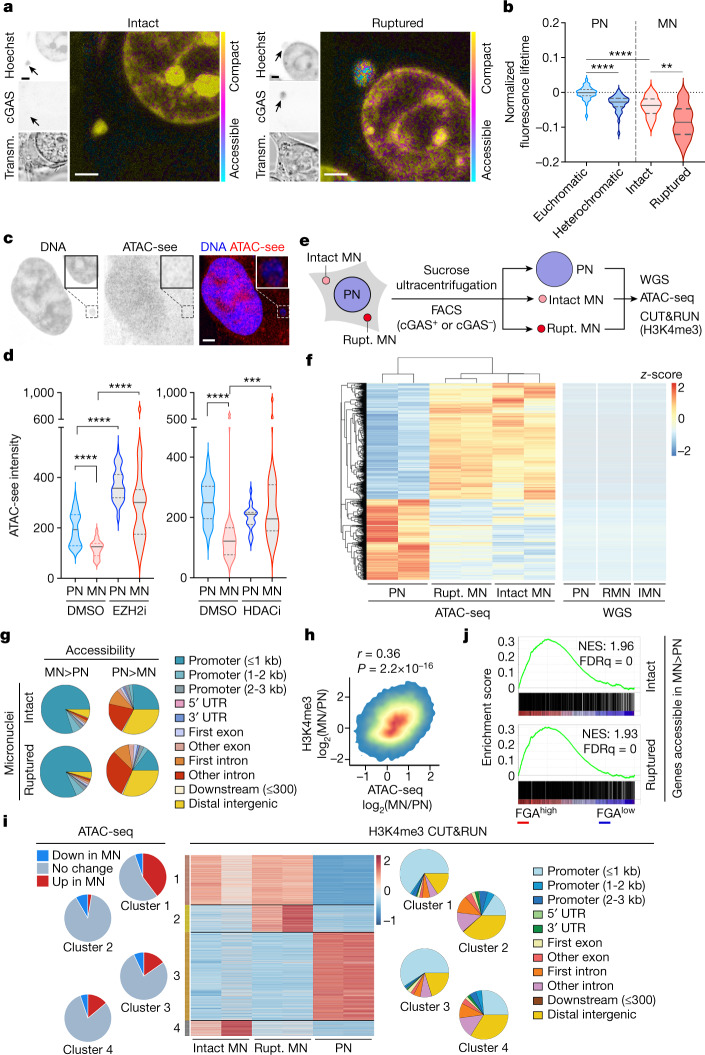
Chromatin accessibility is altered in micronuclei. **a**, Representative fluorescence-lifetime imaging microscopy images from 3 biological replicates of cGAS–GFP-expressing 4T1 cells that express cGAS and green fluorescent protein stained with Hoechst (blue). Images show intact (left) and ruptured (right) cells. Fluorescence lifetime is shown in pseudocolour; scale bar, 2 µm. Transm., transmission light microscopy. **b**, Violin plots denote fluorescence lifetime in euchromatic (Euch.) and heterochromatic (Hetero.) regions of primary nuclei (blue), and intact and ruptured micronuclei of cGAS–GFP-expressing *Trex1*-knockout 4T1 cells.**P < 0.01, ****P < 0.0001, *n* = 3; two-sided Mann–Whitney *U*-test; solid and dotted lines represent the median and quartiles, respectively. **c**, Representative ATAC-see (red) fluorescence images from 3 biological replicates of an MDA-MB-231 cell with a micronucleus stained for DNA (blue); scale bar, 2 µm. **d**, Violin plots representing ATAC-see signal intensity quantification of primary nuclei (PN) and micronuclei (MN) of MDA-MB-231 cells treated with either DMSO, an EZH2 inhibitor (EZH2i) or HDACi; ***P < 0.001, ****P < 0.0001, two-sided Mann–Whitney *U*-test, *n* = 5; solid and dotted bars represent the median and quartiles, respectively. **e**, Schematic of the isolation of micronuclei showing intact and ruptured micronuclei and primary nuclei. **f**, Heat maps showing differentially accessible genomic peaks from ATAC-seq (left) and WGS (right) from primary nuclei, ruptured micronuclei and intact micronuclei isolated from 4T1 cells; *n* = 2. **g**, Pie charts representing positional information of differentially accessible peaks in micronuclei compared with primary nuclei from 4T1 cells; *n* = 2. **h**, Density plot comparing change in H3K4me3 CUT&RUN read counts and ATAC-seq read counts in the same genomic region in micronuclei and primary nuclei from 4T1 cells; comparison performed with two-sided Spearman’s rank correlation statistics; *n* = 2, *r* = 0.36, *P* = 2.2 × 10^−16^. **i**, Heat map (middle) of H3K4me3 CUT&RUN peaks performed on isolated intact and ruptured micronuclei and primary nuclei of 4T1 cells (*n* = 2) revealing 4 clusters. Pie charts denote the differential accessibility of the reads from individual clusters on the heat map in micronuclei and primary nuclei (left), as well as the positional composition of the H3K4me3 of these reads (right). **j**, Enrichment plots of genes with promoters that are more accessible in intact micronuclei (top) or ruptured micronuclei (bottom) compared with primary nuclei in 4T1 cells in comparison to human breast tumours belonging to the top (FGA^high^) or bottom (FGA^low^) quartile of fraction genome altered, according to the TCGA. NES, normalized enrichment score; FDRq, false discovery rate q-value. Source data

**Extended Data Fig. 6:**
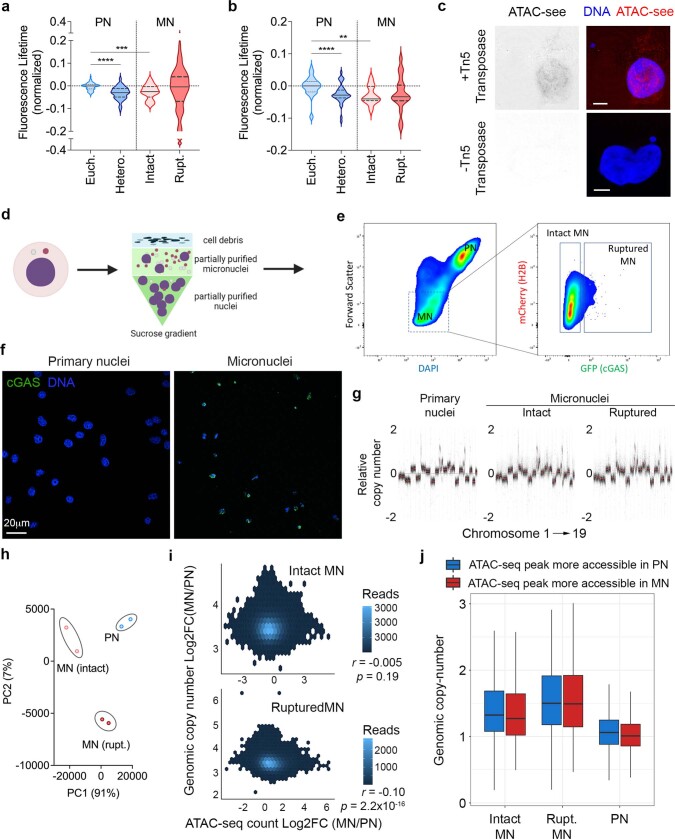
Micronuclei have distinct accessibility as compared with primary nuclei. **a**, Violin plots representing the normalized distribution of fluorescence lifetime in euchromatic (Euch.) and heterochromatic (Hetero.) regions of primary nuclei (PN), ruptured and intact micronuclei (MN) from 4T1 cells, *** *p* < 0.001, **** *p* < 0.0001, n = 73 cells (31 intact and 42 ruptured micronuclei) from five biological replicates, two-sided Mann-Whitney test, solid and dashed bars in the plot represent the median and quartiles, respectively. **b**, Violin plots representing the normalized distribution of fluorescence lifetime in euchromatic (Euch.) and heterochromatic (Hetero.) regions of primary nuclei (PN), ruptured and intact micronuclei (MN) from MDA-MB-231 cells, *** *p* < 0.001, **** *p* < 0.0001, n = 3 biological replicates, two-sided Mann-Whitney test, solid and dashed bars in the plot represent the median and quartiles, respectively. **c**, Representative fluorescence images from 3 biological replicates showing ATAC-see signal in MDA-MB-231 cells treated with (top) or without (bottom) fluorophore-tagged adaptors-loaded Tn5 transposase, scale bar 5 μm. **d**, Experimental schematic for the isolation of primary nuclei, intact micronuclei, and ruptured micronuclei that will be separated by fluorescence activated cell sorting represented in **e**. **e**, Density area plots of a representative micronuclei isolation flow cytometry experiment from 4T1 cells expressing H2B-mCherry and GFP-cGAS, right panel is the subset of the gate applied on the left panel (dashed box). **f**, Representative immunofluorescence image from 3 independent experiments showing isolates of primary nuclei and micronuclei from 4T1 cells after sucrose gradient ultracentrifugation stained for DNA (blue) and cGAS (green), scale bar 20 µm **g**, Relative copy number calling from MoCaSeq pipeline ordered by chromosome (x-axis) from isolated primary nuclei, intact micronuclei, and ruptured micronuclei of 4T1 cells. **h**, Principal component analysis (PCA) plot of intact micronuclei (MN), ruptured (rupt.) MN, and primary nuclei (PN) of 4T1 cells based on peak reads from ATAC-seq, n = 2 biological replicates. **i**, Scatter plot showing the comparison of the log 2 fold change values of genomic copy number vs. log2 fold change of ATAC-seq counts in a given region between intact MN (top) or ruptured MN (bottom) vs. PN of 4T1 cells. Two-sided Pearson’s rank correlation statistics are shown on the panel. **j**, Box plot showing genomic copy number distributions obtained from WGS of intact MN, ruptured MN, and PN of 4T1 cells categorized by differentially accessible regions from ATAC-seq peaks obtained from previous ATAC-seq experiment on MN and PN of 4T1 cells (blue = more accessible in PN, red = more accessible in MN). Line represents median value, n = 2 biological replicates for ATAC-Seq, single replicate for WGS, black line represents median value, the bounds of the box are the interquartile range (Q1 to Q3), the whiskers are defined by 1.5*interquartile range beyond Q1 and Q3, the minimum was 0.1 and the maximum was 3.011. Source data

To test whether abnormalities in histone PTM distribution alter chromatin structure in micronuclei, we used three orthogonal approaches, starting with fluorescence lifetime imaging microscopy (FLIM)^27^, which reliably identified euchromatic and heterochromatic regions in primary nuclei (Fig. 3a). Chromatin in intact micronuclei was compact, resembling heterochromatin (Fig. 3a,b and Extended Data Fig. 6a,b). Chromatin in ruptured micronuclei was compact in MDA-MB-231 cells but displayed a wide distribution in 4T1 cells (Extended Data Fig. 6a,b). We know that this was due to partly digested DNA by cytosolic nucleases because CRISPR–Cas9-mediated knockout^20^ of the nuclease *Trex1* abolished signal heterogeneity in ruptured micronuclei in 4T1 cells (Fig. 3b).

We next treated MDA-MB-231 cells with a transposase loaded with fluorophore-tagged adaptors to image chromatin accessibility at subcellular resolutions, a technique known as assay of transposase-accessible chromatin with visualization (ATAC-see)^28^. The ATAC-see fluorescence signal was markedly diminished in micronuclei but was restored when cells were treated with either EZH2 inhibitors or HDAC inhibitors (Fig. 3c,d and Extended Data Fig. 6c).

Third, we performed an ATAC with sequencing (ATAC-seq), coupled with whole-genome sequencing (WGS), on primary nuclei, intact micronuclei and ruptured micronuclei (Fig. 3e, Extended Data Fig. 6d–g and Methods). Principal component analysis based on merged peaks across samples (*n* = 14,676) indicated differences in overall chromatin accessibility between primary nuclei as well as intact and ruptured micronuclei (Extended Data Fig. 6h). Micronuclei and primary nuclei exhibited a substantial number of differentially accessible peaks (Fig. 3f), and this could not be accounted for by any over- or under-representation of associated genomic regions in micronuclei (Fig. 3f and Extended Data Fig. 6i,j).

**Extended Data Fig. 7:**
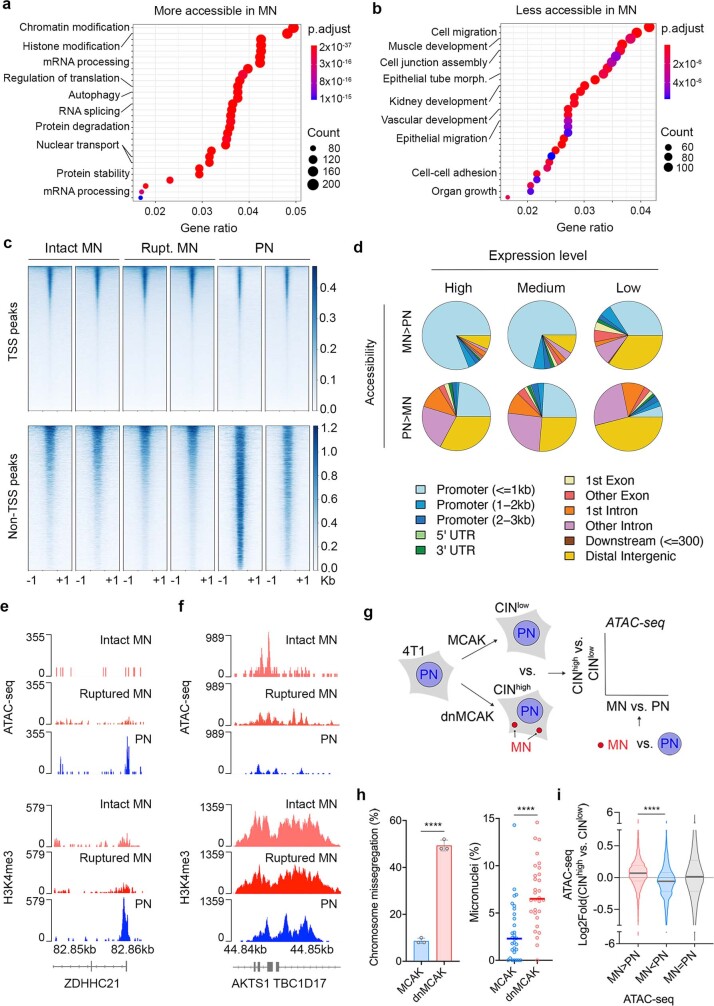
Changes in chromatin accessibility in micronuclei exhibit a positional bias. **a**, Enriched pathways from regions that are more accessible in micronuclei (obtained from ATAC-seq experiment) relative to primary nuclei, sorted by gene ratio. **b**, Enriched pathways from regions that are less accessible in micronuclei (obtained from ATAC-seq experiment) compared with primary nuclei, sorted by gene ratio. For **a** and **b**, pathway enrichment was determined using an over-representation statistical test (a one-sided version of Fisher’s exact test) and p-values were adjusted for multiple hypothesis testing via the Benjamini-Hochberg correction. **c**, Heat map representing chromatin accessibility from ATAC-seq peaks at transcription start sites (TSS peaks) and all other sites (non-TSS peaks) in primary nuclei (PN), intact (Intact MN) and ruptured micronuclei (Rupt. MN) from 4T1 cells, n = 2 biological replicates. **d**, Pie charts showing annotations of genomic regions from ATAC-seq experiment that are more accessible in MN (left charts) and less accessible in MN (right charts), stratified by the baseline expression of the genes within that region obtained from RNA-sequencing experiment of 4T1 dnMCAK CIN^high^ cells (top = high expression, middle = medium expression, bottom = low expression). **e**-**f**, Sequencing tracks in ZDHHC21 (d), AKTS1, and TBC1D17 (e) promoter loci in intact MN, ruptured MN, and PN of 4T1 cells, showing ATAC-seq peaks (top panel) and H3K4me3 CUT&RUN peaks (bottom panel). **g**, Experimental strategy schematic depicting the generation of 4T1 MCAK (CIN^low^) and dnMCAK (CIN^high^) cells, which are then subjected to ATAC-seq to compare their chromatin accessibility to the chromatin accessibility results obtained from ATAC-seq experiments on isolated micronuclei (MN) and primary nuclei (PN) of 4T1 cells. **h**, CIN profiling on 4T1 MCAK (CIN^low^) and dnMCAK (CIN^high^) cells. Left panel shows chromosomal missegregation percentage out of 50 cells per biological replicate in anaphase, **** *p* < 0.0001, two-sided *t-*test, bars represent mean ± SD, n = 3 biological replicates, 150 total number of anaphase cells observed. Right panel shows micronuclei percentage over primary nuclei in a field of view in 63x magnification, **** *p* < 0.0001, two-sided Mann-Whitney test, bars represent median, n = 3 biological replicates, 30 fields of view. **i**, Violin plots representing log2 fold change of ATAC-seq peaks from 3 biological replicates comparing 4T1 CIN^high^ to 4T1 CIN^low^ cells. The peaks are categorized by their relative accessibility in micronuclei vs. primary nuclei purified from 4T1 cells. The categories are: 1) peaks that are more accessible in MN vs. PN (MN>PN), 2) peaks that are more accessible in PN vs. MN (MN<PN), 3) and peaks that are equally accessible in MN vs. PN (MN = PN), n = 3 biological replicates, **** p < 0.0001, two-sided Mann-Whitney test, solid and dashed bars in the plot represent the median and quartiles, respectively. Source data

Gene-set enrichment analysis (GSEA) revealed that genes that were preferentially accessible in micronuclei as opposed to primary nuclei were involved in chromatin and histone modification, RNA biogenesis and processing, and protein turnover (Extended Data Fig. 7a). Conversely, genes that were less accessible in micronuclei were related to developmental pathways, cellular adhesion and migration (Extended Data Fig. 7b). Surprisingly, differential chromatin accessibility in micronuclei involved a profound positional bias; whereas many transcription start sites (TSS) were more accessible in micronuclei, non-TSS peaks were less accessible (Extended Data Fig. 7c). Deeper positional analysis revealed an overwhelming predisposition towards more-accessible promoters, and their associated gene bodies in micronuclei, which was consistent across highly transcribed and poorly transcribed genes. By contrast, there was reduced accessibility within intronic and distal intergenic regions (Fig. 3g, Extended Data Fig. 7d and Supplementary Fig. 3).

We investigated whether the unexpected differences in promoter accessibility might be explained by the aberrant deposition of H3K4me3 that marks transcriptionally active promoters, and that was biochemically enriched in micronuclei (Extended Data Fig. 3b). CUT&RUN analysis revealed a positive correlation between relative H3K4me3 enrichment in micronuclei and associated changes in ATAC-seq signal intensity (Fig. 3h and Extended Data Fig. 7e, f). Further analysis identified four clusters of differential H3K4me3 enrichment in micronuclei, one of which (cluster 1) exhibited increased H3K4me3 deposition near the promoter regions in micronuclei and a corresponding increase in chromatin accessibility (Fig. 3i).

## Heritable epigenetic abnormalities from micronuclei

To determine whether changes in chromatin accessibility in micronuclei reflect the global epigenetic landscape for chromosomally unstable cells, we generated otherwise isogenic pairs of 4T1 cells with different levels of CIN (CIN^high^ and CIN^low^) using active and dominant-negative forms of the kinesin-13 protein MCAK, as previously described^11,29^ (Extended Data Fig. 7g). These isogenic cell pairs contained different rates of chromosome missegregation and micronuclei (Extended Data Fig. 7h). Regions that were more accessible in micronuclei than in primary nuclei were also more accessible in CIN^high^ tumour cells than in their CIN^low^ counterparts. The opposite was also true (Extended Data Fig. 7i). This trend remained consistent when performing the same analysis on the TSS regions of the genome (data not shown). We tested whether genes that were more accessible in micronuclei than in primary nuclei were transcriptionally enriched in human cancers with CIN by comparing human breast tumours in the top and bottom thirds of the fraction of genome altered (FGA) according to The Cancer Genome Atlas (TCGA). There was significant transcriptional enrichment in micronuclei-accessible genes in FGA^high^ tumours. Similarly, transcripts of genes that were less accessible in micronuclei were correspondingly depleted in FGA^high^ relative to FGA^low^ tumours (Fig. 3j, Supplementary Table 4 and Supplementary Fig. 3).

**Fig. 4:**
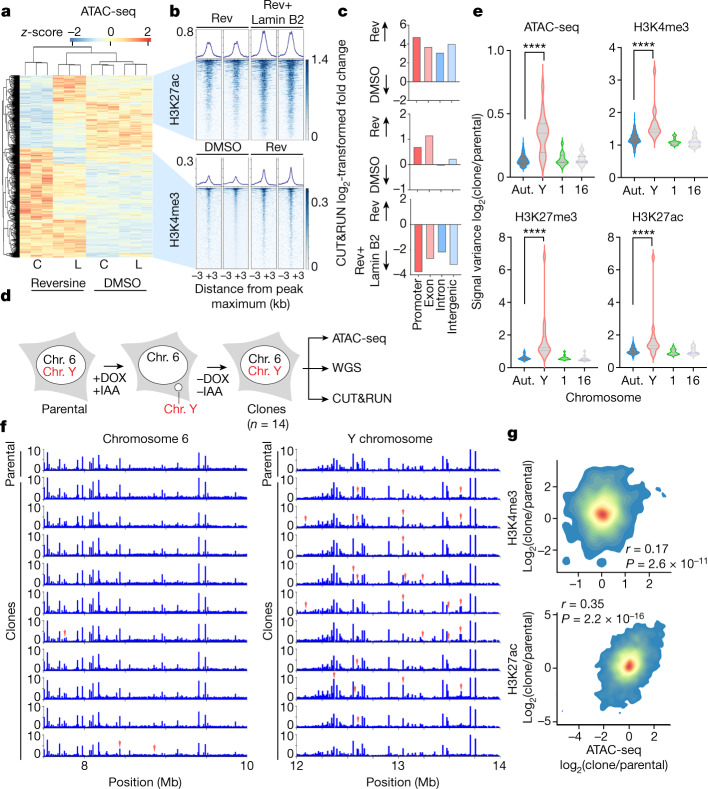
CIN drives long-term changes in chromatin accessibility. **a**, Heat map representing chromatin accessibility from ATAC-seq in control (C) or lamin-B2-overexpressing (L) *TP53*-knockout RPE-1 cells treated with reversine or DMSO; *n* = 3. **b**, Tornado plots from CUT&RUN analysis representing H3K27ac- and H3K4me3-bound chromatin in regions corresponding to the accessibility heat map shown in **a**; *n* = 3.Scales on the right side signify peak intensity. **c**, Log_2_-transformed fold change in the number of H3K4me3 (top), H3K27me3 (middle) and H3K27ac (bottom) CUT&RUN peaks on long-term reversine-treated (Rev) versus DMSO-treated *TP53*-knockout RPE-1 cells and those expressing lamin B2; graphs show pairwise comparisons. **d**, Experimental schematic representation depicting CEN-SELECT DLD-1 cell system used to generated Y-chromosome missegregation. Chromosome (Chr.) 6 is an example of a control autosome. **e**, Violin plots representing intraclonal variance from ATAC-seq, H3K4me3 CUT&RUN, H3K27me3 CUT&RUN and H3K27ac CUT&RUN across 10-kb segments in each of 14 DLD-1 clones. Aut., autosomes; Y, Y chromosome, Chr. 1 and Chr. 16. *****P < 0.00001, two-sided Mann–Whitney *U*-test, *n* = 14 clones of CEN-SELECT DLD-1 cells. **f**, Genome viewer plot showing copy-number-normalized ATAC-seq counts in a 2-kb region of chromosome 6 (control autosome) and the Y chromosome of DLD-1 CEN-SELECT parental control cells and the individual clones. Top row represents parental cells that did not undergo missegregation; the remaining rows represent single cell clones that underwent missegregation and transient micronucleation of the Y chromosome. Red arrows denote differentially accessible peaks. **g**, Density plot showing the comparison of the log_2_-transformed fold change in H3K4me3 CUT&RUN reads (top; *r* = 0.17, *P* = 2.6 × 10^−11^) or H3K27ac CUT&RUN read counts (bottom; *r* = 0.35, *P*= 2.2 × 10^−16^) against ATAC-seq read counts in a given region between DLD-1 CEN-SELECT clones or parental cells; two-sided Spearman’s rank correlation statistics. Source data

**Extended Data Fig. 8:**
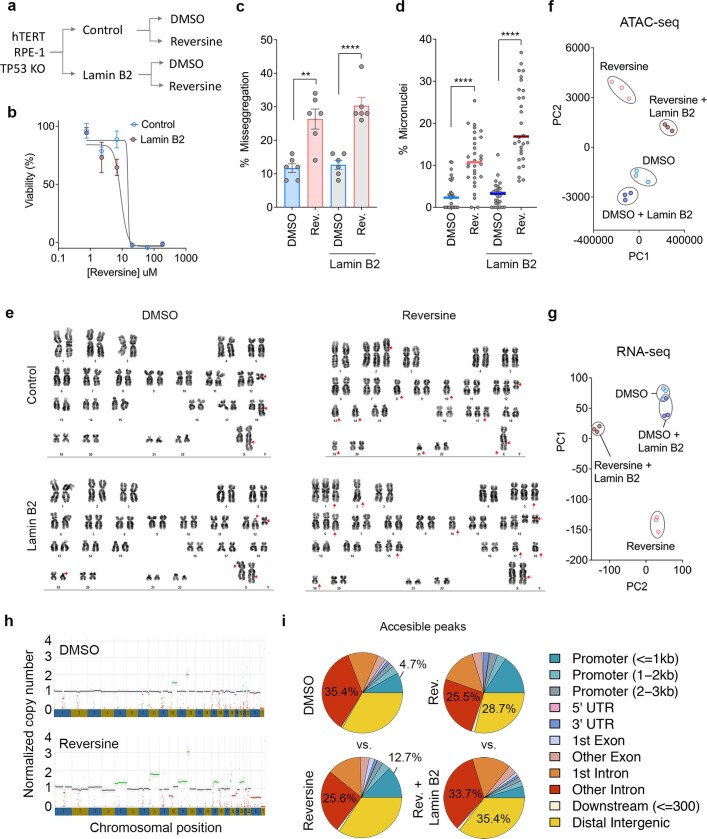
The generation of isogenic hTERT RPE-1 cell lines with different CIN rates. **a**, Experimental schematic depicting the generation of isogenic hTERT p53 KO RPE-1 cells with different CIN rates. **b**, IC50 curves of reversine-treated control and Lamin B2 overexpressing RPE-1 cells, points represent mean ± SD, n = 3 biological replicates. **c**, Percentage of cells undergoing anaphase with chromosome mis-segregation in control or lamin B2 overexpressing RPE-1 cells long-term-treated vehicle (DMSO) or reversine (Rev.); n = 300 cells per condition, 6 biological replicates, *** *p* < 0.001, two-sided *t-*test, bars represent mean ± SD. **d**, Percentage of micronuclei relative to primary nuclei in control or lamin B2 overexpressing RPE-1 cells long-term-treated with vehicle (DMSO) or reversine (Rev.); n = 30 fields of view in 63x magnification, 3 biological replicates, **** *p* < 0.0001, two-sided *t-*test, bars represent median. **e**, Conventional karyotypes of control or lamin B2 overexpressing RPE-1 cells long-term-treated with vehicle (DMSO) or reversine, red arrows denote chromosomal abnormalities. **f**, Principal component analysis (PCA) plot of TP53 KO hTERT-RPE-1 cells based on accessibility from ATAC-seq, n = 3 biological triplicates. **g**, Principal component analysis (PCA) plot of TP53 KO hTERT-RPE-1 cells based on RNA expression obtained from RNA-seq, n = 3 biological triplicates. **h**, Normalized copy number (CN) calling results from GATK4 pipeline in RPE-1 cells undergoing long-term DMSO treatment (top) or long-term reversine treatment (bottom). Red line indicates CN loss, green line indicates CN gain, grey line indicates no CN change. **i**, Pie charts showing genomic region annotations of accessible peaks in RPE-1 p53 KO cells undergoing long-term DMSO treatment vs. long-term reversine treatment (left panels) and reversine vs. reversine + lamin B2 overexpression (right panels). Source data

**Extended Data Fig. 9:**
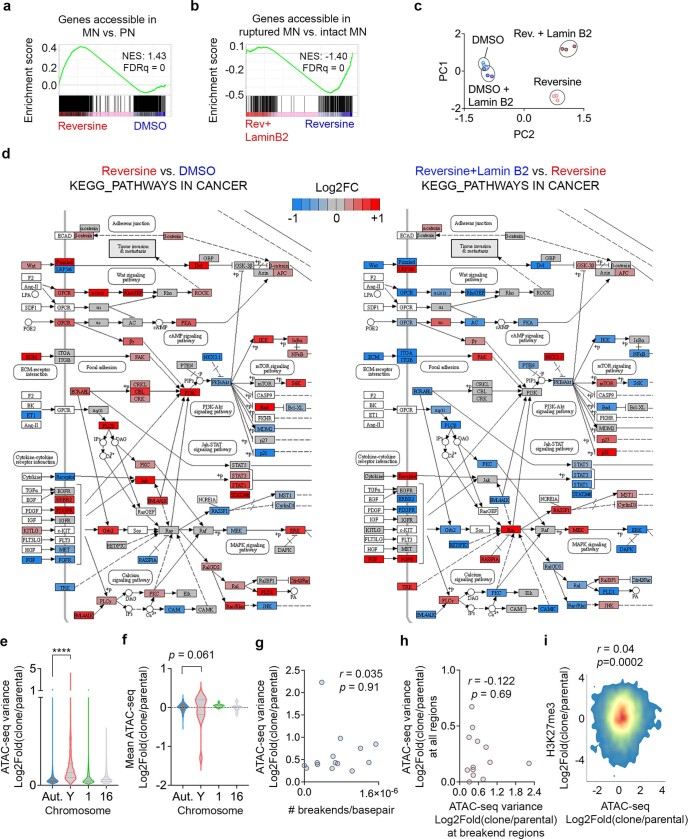
Chromosome missegregation upregulates cancer pathways and heterogeneity in genomic accessibility. **a**, Enrichment plots of genes that are more accessible in MN vs. PN of 4T1 cells against genes that are more accessible in in either long-term reversine-treated or DMSO-treated p53 KO RPE-1 cells. **b**, Enrichment plots of genes that are more accessible in ruptured MN vs. PN of 4T1 cells against genes that are more accessible in either long-term reversine-treated p53 KO RPE-1 cells or reversine-treated p53 KO RPE-1 cells overexpressing lamin B2. **c**, Principal component analysis (PCA) plot of TP53 KO hTERT-RPE-1 cells based on a list of tumor suppressor genes and oncogenes, n = 3 biological triplicates. **d**, KEGG pathway analysis from KEGG Pathways in cancer – Homo sapiens (human) database (map05200). Long-term reversine-treated vs. DMSO-treated p53 KO RPE-1 cells analysis is shown on the left, while long-term reversine-treated lamin B2-overexpressing p53 KO RPE-1 cells vs. long-term reversine-treated p53 KO RPE-1 cells is shown on the right. **e**, Violin plots representing intraclonal variance across 10kb segments in each of 14 DLD-1 CEN-SELECT clones. Aut. = autosomes, Y = Y chromosome, 1 = chromosome 1, 16 = chromosome 16. Distribution represents 86,666 calculated values for autosomes, 730 calculated values for Y chromosome. ***** *p* < 0.0001, two-sided Mann-Whitney test. **f**, Inter-clonal comparison of fold change of ATAC-seq peaks in autosomes, and the Y chromosomes in 14 DLD-1 single clones isolated from CEN-SELECT system to parental control. Aut. = autosomes, Y = Y chromosome, 1 = chromosome 1, 16 = chromosome 16. Bars represent median, statistical significance tested using two-sided Mann-Whitney test. **g**, Scatter plot comparing ATAC-seq fold change variance to number of breakends per basepair in the Y-chromosome of the clones of DLD-1 CEN-SELECT system cells. **h**, Scatter plot comparing ATAC-seq fold change variance in regions with breakends to ATAC-seq fold change variance across all regions in the Y-chromosome of the clones of DLD-1 CEN-SELECT system cells. For **g** and **h**, statistical test was done using two-sided Pearson’s rank correlation. No adjustments were made for multiple comparisons. **i**, Density plot showing the comparison of the log2 fold change of H3K27me3 CUT&RUN reads vs. ATAC-seq reads in a given region between DLD-1 CEN-SELECT clones vs. parental, correlation measured with two-sided Spearman’s rank correlation statistic. Source data

Next, we subjected control and lamin-B2-expressing chromosomally stable *TP53*-knockout RPE-1 cells to long-term (8 passages over about 2 months) treatment with reversine, an MPS1 inhibitor that induces chromosome segregation defects^30^, at sublethal doses (Extended Data Fig. 8a,b). Reversine-treated cells exhibited an increase in anaphase chromosome missegregation, micronuclei formation, and pervasive numerical and structural chromosomal aberrations indicative of CIN (Extended Data Fig. 8c–e). In line with previous reports^31^, around 45% of micronuclei were successfully reincorporated at the first mitotic division (Supplementary Fig. 4). We performed WGS, ATAC-seq and RNA sequencing (RNA-seq) 48 h after the last treatment with reversine, and we observed global changes in both chromatin accessibility and transcription (Fig. 4a and Extended Data Fig. 8f,g), which again could not be explained by genomic copy-number alterations (Extended Data Fig. 8h). Lamin B2 on its own did not meaningfully affect chromatin structure or transcription but it partly rescued some of the changes induced by reversine (Fig. 4a, Extended Data Fig. 8f,g and Supplementary Table 5). There was an increased accessibility at promoter regions in reversine-treated cells (Extended Data Fig. 8i). Genes that were generally more accessible in micronuclei were also transcriptionally enriched in reversine-treated cells, whereas those that were exclusively accessible in ruptured micronuclei were correspondingly downregulated on lamin B2 overexpression (Extended Data Fig. 9a,b). Many of the differentially expressed genes belonged to oncogenic or tumour-suppressive pathways, and treatment of RPE-1 cells with reversine upregulated oncogenic transcriptional programs that were rescued by the overexpression of lamin B2 (Extended Data Fig. 9c,d and Supplementary Table 6). We next performed CUT&RUN on reversine-treated RPE-1 cells, profiling H3K4me3-, H3K27me3- and H3K27ac-bound chromatin. H3K4me3 was enriched in peaks that became more accessible after treatment with reversine, whereas H3K27ac was correspondingly enriched upon lamin B2 overexpression selectively in peaks with accessibility that was increased by reversine but simultaneously rescued by lamin B2 overexpression (Fig. 4b,c).

To examine whether CIN-induced epigenetic abnormalities are specific to chromosomes that transit in micronuclei, we used three orthogonal approaches. Starting with the inducible Y-chromosome-specific missegregation system, we isolated single-cell-derived clones in which the Y chromosome has been stably reincorporated into the primary nucleus after micronucleation^18^. By performing WGS, ATAC-seq, and CUT&RUN (of H3K4me3-, H3K27me3- and H3K27ac-bound chromatin) on the parental cells as well as 14 clones, we were able to assess the long-term epigenetic consequences that are specific to the missegregated Y chromosome in each clone compared with the remaining normal autosomes, as well as to the parental line (Fig. 4d). ATAC-seq read counts along the genome were normalized to DNA copy number obtained from WGS of each clone^18^ (Methods and Supplementary Figs. 5 and 6). Relative to the parental cells, there was increased interclonal variability in the copy-number-corrected accessibility profile and histone mark distribution in the Y chromosome but not in the autosomes or the X chromosome (Fig. 4e,f and Extended Data Fig. 9e,f). Variance in chromatin accessibility along 10-kilobase (kb)-long genomic segments on the Y chromosome in individual clones also greatly exceeded that of the autosomes and the X chromosome (Extended Data Fig. 9e). Importantly, ATAC-seq signal abnormalities did not correlate with chromosomal breaks or rearrangements (Extended Data Fig. 9g, h). Although there was no consistent directionality in chromatin accessibility changes (that is, some clones exhibited increased accessibility whereas others displayed reduced accessibility in the same genomic region of the Y chromosome compared with the parental line), the deviation in ATAC-seq signal mirrored corresponding changes in H3K4me3 and H3K27ac (but not H3K27me3) occupancy in individual clones (Fig. 4g, Extended Data Fig. 9i and Supplementary Fig. 7).

**Extended Data Fig. 10:**
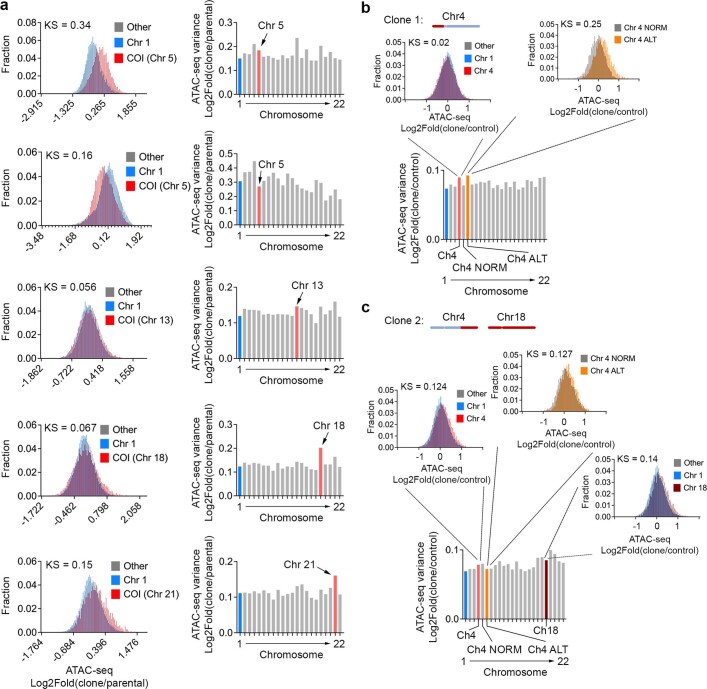
Chromosome missegregation and micronucleation promotes epigenetic dysregulation. **a**, (Left) Histogram of log2 fold change of ATAC-seq counts between MMCT HCT116 cells’ clones compared to parental for chromosome of interest (COI), chromosome 1 (Chr 1; as a single chromosome control), and all other chromosomes combined (Other). KS = Kolmogorov-Smirnov test’s absolute maximum distance value between COI vs. other chromosomes. (Right) The variance of the log 2 fold change values of ATAC-seq counts of clone vs. parental control in a particular chromosome. Chromosome of interest is colored in red while chromosome 1 is colored in blue, n = 3 biological replicates. **b**, Analysis of clone 1 of chr 4 missegregation system in RPE-1 cells. Altered region is represented by the chromosome diagram on the top (red = altered, blue = normal). The bar plot represents the variance of log2 fold change of ATAC-seq counts (single cell-derived clones derived after transfection of p53 KO RPE-1 cells with chromosome 4 targeting sgRNA or non-targeting control) in a particular chromosome. Chromosome 1 is shown in blue (Chr 1), the whole chromosome 4 (Ch4) is shown in red, altered region of chromosome 4 is shown in gold (Ch4 ALT), and unaltered chromosome 4 region is labeled as Ch4 NORM. Left inset shows the histogram of log2 fold change of ATAC-seq counts between clone 1 compared to parental for whole chromosome 4 (red), chromosome 1 (blue) or other chromosomes (gray). Right inset shows the histogram of log2 fold change of ATAC-seq counts between clone 1 compared to parental for altered region of chromosome 4 (gold) and the unaltered region of chromosome 4 (gray). KS = Kolmogorov-Smirnov test’s absolute maximum distance value between chromosome 4 values vs. other chromosomes (left inset) or between altered chromosome 4 values vs. unaltered chromosome 4 (right inset). **c**, Analysis of clone 2 of chr 4 missegregation system in RPE-1 cells. Altered region is represented by the chromosome diagram on the top (red = altered, blue = normal), note that chromosome 18 is also affected due to spontaneous duplication. The bar plot represents the variance of log2 fold change of ATAC-seq counts (single cell-derived clones derived after transfection of p53 KO RPE-1 cells with chromosome 4 targeting sgRNA or non-targeting control) in a particular chromosome. Chromosome 1 is shown in blue (Chr 1), the whole chromosome 4 (Ch 4) is shown in red, altered region of chromosome 4 is shown in gold (Ch4 ALT), unaltered chromosome 4 region is labeled as Ch4 NORM, and chromosome 18 is shown in brown. Left inset shows the histogram of log2 fold change of ATAC-seq counts between clone 2 compared to parental for whole chromosome 4 (red), chromosome 1 (blue) or other chromosomes (gray). Middle inset shows the histogram of log2 fold change of ATAC-seq counts between clone 2 compared to parental for altered region of chromosome 4 (gold) and the unaltered region of chromosome 4 (gray). Right inset shows the histogram of log2 fold change of ATAC-seq counts between clone 2 compared to parental for chromosome 18 (brown), chromosome 1 (blue) or other chromosomes (gray). KS = Kolmogorov-Smirnov test’s absolute maximum distance value between chromosome 4 values vs. other chromosomes (left inset), between altered chromosome 4 values vs. unaltered chromosome 4 (middle inset), or between chromosome 18 vs. other chromosomes (right inset). Source data

We next used microcell-mediated chromosome transfer in HCT116 cells, a diploid colorectal cancer-derived cell line, to generate clones with specific trisomies, including chromosomes 5, 13, 18 and 21, as previously described^32^. In this artificial system, the transferred chromosome transited through a micronucleus-like structure before being incorporated into the acceptor cell, leading to the establishment of a single-cell-derived clonal population containing the third copy of the transferred chromosome. It is important to note that unlike the Y chromosome, of which there is one copy, analysis of trisomic clones probably underestimates abnormalities in chromatin accessibility arising from the single missegregated copy. We observed changes in genomic copy-number-normalized chromatin accessibility in clones harbouring three trisomic chromosomes (chromosomes 5, 18 and 21), and these changes were restricted to the trisomic chromosome as demonstrated either by alterations in relative chromatin accessibility or in the ATAC-seq signal variance in the clone compared with the parental diploid population (Extended Data Fig. 10a). The epigenetic abnormalities seen in two independent clones harbouring trisomy 5 were similar, despite only one of them undergoing chromothripsis^32^ (Extended Data Fig. 10a, second clone).

**Fig. 5:**
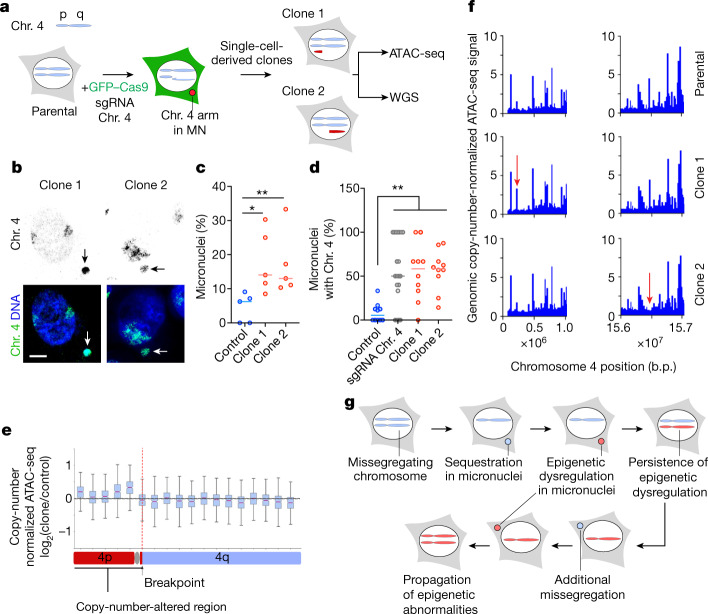
Chromosomal transit in micronuclei promotes heritable epigenetic abnormalities. **a**, Experimental schematic depicting missegregation of Chr. 4 in RPE-1 *TP53*-knockout cells induced using a chromosome 4 telomeric CRISPR guide; sgRNA, single guide RNA. **b**, Representative chromosome paint images from 2 biological replicates in RPE-1 *TP53*-knockout single-cell clones from a chromosome-4 missegregation system showing chromosome 4 (green) and DNA (blue). Scale bar, 5 µm. **c**, Percentage of micronuclei in single-cell-derived clones derived after transfection of *TP53*-knockout RPE-1 cells with either chromosome-4-targeting sgRNA or non-targeting control sgRNA; *P < 0.05, **P < 0.01, two-sided Mann–Whitney *U*-test; bars represent medians; *n* = 5 fields of view under 63× magnification. **d**, Percentage of micronuclei that contain chromosome 4 in single-cell-derived clones derived after transfection of *TP53*-knockout RPE-1 cells with chromosome 4 targeting sgRNA or non-targeting control or the mixed cell population shortly after transfection with the chromosome 4 targeting sgRNA; **P < 0.01, two-sided Mann–Whitney *U*-test; bars represent median; *n* = 10 fields of view under 63× magnification. **e**, Box plot (10-Megabase bin) showing copy-number normalized change of ATAC-seq counts on chromosome 4 between clone 1 and the control clone treated with the non-targeting guide sgRNA. The line represents the median and error bars represent minimum and maximum values; *n* = 3; solid red line represents the median; the bounds of the box are the interquartile range (Q1 to Q3); error bars are defined by 1.5*interquartile range beyond Q1 and Q3. **f**, Genome viewer plot showing copy-number-normalized ATAC-seq counts in the region of chromosome 4 that is copy-number altered in clone 1 (left) or clone 2 (right). Red arrows denote differentially accessible peaks compared with the control clone. **g**, Model showing how continuous chromosomal missegregation followed by micronucleation can introduce heritable epigenetic dysregulation. Because chromosomally unstable cells tend to continuously undergo chromosomal missegregation, this could in turn propagate epigenetic abnormalities through the same mechanism, leading to epigenetic heterogeneity. Source data

Finally, we induced partial chromosome missegregation using ribonucleoprotein transfection of CRISPR–Cas9 single-guide RNA complexes targeting chromosome 4 in *TP53*-knockout RPE-1 cells, as previously described^33^ (Fig. 5a). In this system, a portion of chromosome 4 underwent micronucleation^33^, and most of the micronuclei formed contained chromosome 4 genetic material (Fig. 5b–d). We generated two clones that underwent spontaneous reincorporation of the missegregated portion of chromosome 4 and, as such, contained partial chromosome 4 copy-number alterations: trisomy within the 4p and 4q arms (Fig. 5a–d). In the second clone, there was also a spontaneous trisomy of chromosome 18. We analysed copy-number-normalized changes in chromatin accessibility along chromosome 4 and observed abnormalities that were restricted to the portion that underwent missegregation in each of the two clones (along with chromosome 18 in clone 2) compared with the same region in control clones with non-targeting guides (Fig. 5e,f and Extended Data Fig. 10b,c). This included abnormalities in the ATAC-seq signal on one side of the CRISPR-induced breakpoint where the missegregation occurred, as shown by the appearance or disappearance of ATAC-seq peaks (Fig. 5e,f).

## Discussion

Our work demonstrates that the transient sequestration of chromosomes in micronuclei disrupts chromatin organization, leading to heritable epigenetic dysregulation long after the chromosome is reintegrated into the primary nucleus. Thus CIN can drive epigenetic reprogramming in cancer by altering the chromatin organization of entire chromosomes in the span of a single cell cycle. On the population level, the continuous formation and reincorporation of micronuclei throughout successive cell divisions steadily contributes to epigenetic abnormalities, promoting cell-to-cell variability in chromatin accessibility (Fig. 5g). As well as serving as a substrate for natural selection, CIN-induced epigenetic reprogramming could lead to dosage compensation^34^, in addition to the well-characterized protein-level compensation^35,36^, to buffer deleterious alterations in chromosome copy number.

The compact chromatin organization in micronuclei is driven by the relative enrichment of stable heterochromatin-associated histone marks and the loss of the more dynamic histone PTMs, such as H3K27ac. Nonetheless, the surprising positional accessibility bias seen near promoter regions raises the possibility that micronuclei might promote transcriptional redistribution, rather than indiscriminate silencing. Under appropriate selective pressures, enrichment of H3K4me3 at certain accessible promoters could serve as a mechanism to enhance the expression of oncogenes or to form circular extrachromosomal DNA, which has an accessible chromatin state and a high density of oncogenes^37–39^. Our data do not definitively rule out the possibility that increased promoter accessibility could be the result of absent transcriptional machinery at the TSS of highly transcribed genes. However, the differential enrichment of H3K4me3, and the observation that increased promoter accessibility in micronuclei is seen in genes with varying expression levels, make this proposition less favoured. Finally, changes in histone PTMs in cytosol-accessible chromatin may also act as a biochemical signal that affects downstream processes such as senescence, the sensing of cytosolic double-stranded DNA by the cGAS–STING pathway, and DNA repair in micronuclei.

## Methods

### Cell culture

Cell lines (MDA-MB-231, 4T1 and RPE-1) were purchased from the American Type Culture Collection (ATCC). *TP53*-knockout MCF10A, *TP53*-knockout RPE-1 and *Trex1* knockout 4T1 cells were gifts from the Maciejowski laboratory at the Memorial Sloan Kettering Cancer Center (MSKCC). OVCAR-3 cells were a gift from J. D. Gonzales. All gifted cells had been obtained from the ATCC. Catalogue numbers from the ATCC are as follows: MDA-MB-231, HTB-26; 4T1, CRL-2539; RPE-1, CRL-4000; MCF10A, CRL-103172; and OVCAR-3, HTB-161. MDA-MB-231 and RPE-1 cells were cultured in DMEM (Corning), and 4T1 and OVCAR-3 cells were cultured in RPMI (Corning); both media were supplemented with 10% FBS and 50 U ml^−1^ penicillin–streptomycin. All cells were periodically tested for mycoplasma contamination.

### Immunofluorescence microscopy and histone PTM quantification

Cells were grown on coverslips until 80–90% confluent. Cells were fixed using ice-cold (−20 °C) methanol for 15 min in the freezer. Cells were then permeabilized with 0.5% Triton-X-100 in bovine serum albumin (TBS-BSA) 1% solution for 5 min at room temperature. Subsequently, cells were washed with TBS-BSA (1%) and incubated for 5 min at room temperature. For samples from patients with HGSOC, specimens were collected as previously reported^40^. All protocols for collection were approved by the institutional review board of MSKCC. Patients gave consent according to the institutional review board-approved standard operating procedures for informed consent. Written informed consent was obtained from all patients before conducting any study-related procedures. The study was conducted in accordance with the Declaration of Helsinki and the good clinical practice guidelines. Antibodies (see Supplementary Table 1 for a complete list and dilutions) were diluted with 1% BSA-TBS solution and incubated at 4 °C overnight. The stain DAPI (0.5 µg ml^−1^) was then added along with secondary antibodies diluted in TBS-BSA (1%). Cells were mounted with Fluoro-Gel with Tris buffer (Electron Microscopy Sciences). Images were collected as described below (super-resolution immunofluorescence microscopy section). Histone PTM positive/negative quantification was done in triplicates for each histone PTM, with 100 micronuclei counted per replicate. Histone PTM intensity quantification was done using ZEN 2 (blue edition) software, for which the fluorescence intensity of the corresponding PTM is normalized to the fluorescence intensity of the DAPI channel.

### Immunoblots

#### Western blots

Cells were lysed with 1× RIPA buffer (EMD Millipore), kept on ice and vortexed 3 times in 10-min intervals. The lysates were then centrifuged at 10,000 rpm for 10 min and debris was discarded. Protein was quantified using the DC protein assay (Bio-Rad) and equal amounts of protein were loaded in each SDS–PAGE gel well and run at 120 V for 1 h. Proteins were then transferred to a nitrocellulose membrane using the standard protocol in the Bio-Rad Trans-Blot Turbo transfer system. The membrane was blocked using TBS blocking buffer (Li-Cor) incubated with primary antibodies (Supplementary Table 2) overnight at 4 °C. Secondary antibodies were incubated for 1 h at room temperature. Western blot membrane scanning was done using an Odyssey CLx system (Li-Cor). For dot blots, isolated nuclei were lysed with 1× RIPA buffer (EMD Millipore), kept on ice and vortexed 3 times in 10-min intervals. The lysates were then centrifuged at 10,000 rpm for 10 min and debris was discarded. No quantification was done for dot blots; an approximation of a 1:20 ratio between micronuclei and primary nuclei was used instead. Then 2 µl of the RIPA extract of the fraction of either the micronucleus or the primary nucleus was blotted on top of a nitrocellulose membrane and air-dried for at least 1 h. The membrane was then blocked using TBS blocking buffer (Li-Cor) incubated with primary antibodies (Supplementary Table 2) overnight at 4 °C. Secondary antibodies were incubated for 1 h at room temperature. Scanning was done using the Odyssey CLx system (Li-Cor) and quantification was done using ImageStudio software (Li-Cor). Antibodies used for immunoblots are listed in Supplementary Table 2. Full uncropped membranes are shown in Supplementary Fig. 1.

### Drug treatments

Cells were treated with reversine (Cayman Chemical Company) at a concentration of 0.5 µM for either 24 h (for MDA-MB-231) or 48 h (4T1). The HDAC inhibitor vorinostat (Sigma-Aldrich; also known as SAHA) was used at a concentration of 0.5 µM for 24 h, and the methyltransferase inhibitor GSK-126 (XcessBio; EZH2) was used at a concentration of 5 µM for 5 days. Long-term reversine treatment was done 24 h after cells were passaged, and the medium was replaced with normal cell medium after 48 h. GSK923295 (Selleck Chemicals) was used at the concentration of 50 nM for 24 h. DMSO was used as the vehicle control.

### Plasmids and molecular biology

Expression of H2B-mCherry and GFP–cGAS was done using plasmids obtained from the Maciejowski laboratory as previously described^20^. Puromycin at the concentration of 2 µg ml^−1^ was used to select for cells expressing both proteins, and subsequently fluorescence-activated cell sorting (FACS) was used to enrich for cells expressing the respective proteins. Lami-B2-mCherry overexpression was achieved using a plasmid obtained from the Hetzer laboratory, as previously described^7^. Blasticidin was used at the concentration of 10 µg ml^−1^ to select for cells overexpressing lamin B2. Cells were transduced using a standard lentiviral protocol (H2B-mCherry and GFP–cGAS) or retroviral protocol (lamin B2-mCherry) using HEK-293T cells to produce the lentivirus and retrovirus vectors. After viral transduction, cells were cultured for 24 h before selection was performed.

### siRNA transfection

We plated 500,000 cells for each condition in a 6-well plate containing a coverslip for each well and allowed to adhere overnight. The next day, cells were transfected with 30 pmol of small interfering RNA (siRNA) anti-lamin A (pre-designed siRNA, ThermoFisher) or its selected negative control (ThermoFisher) following the standard protocol for lipofectamine RNAiMAX (ThermoFisher). After 48 h the cells were lysed with RIPA buffer, and the lysate subjected to western blot was analysed using anti-lamin A antibody (Thermo Scientific) to test for efficient knockdown. Coverslips were fixed and stained as previously described in the 'Immunofluorescence microscopy and histone PTM quantification' section.

### Fluorescence lifetime imaging microscopy

#### Microscopy

Fluorescence lifetime images were collected with a Zeiss LSM 880 (Carl Zeiss Microscopy) microscope equipped with a Spectra-Physics MaiTai HP laser (Spectra-Physics) for two-photon excitation, tuned at 800 nm, and collected using a Zeiss 63×/1.41 NA oil objective. The fluorescence signal was collected by a photomultiplier tube (H7422P-40, Hamamatsu) and recorded using a FLIMbox (model A320 ISS) to obtain the lifetime information. The pixel dwell time for the acquisitions was 16 μs and the images were taken with sizes of 256 × 256 pixels, accumulating 15 frames. The data from each pixel were recorded and analysed using the SimFCS software (Laboratory for Fluorescence Dynamics, University of California, Irvine, CA) to perform the phasor transformation and with custom MATLAB code to quantify the lifetime. The phasor position was corrected for the instrument response function by acquiring the fluorescence of a solution with a known lifetime (0.4 mM Coumarin 6 dissolved in ethanol, *τ* = 2.5 ns). The lifetime was determined as *τ* = *s*/*ωg*, where *τ* is the fluorescence lifetime, *g* and *s* are the phasor coordinates and *ω* is the laser frequency (80 MHz).

#### Cell labelling and image processing

The samples were labelled with one drop per well of Hoechst 33342 (NucBlue, Thermofisher Scientific) and imaged after 20 min of incubation. For every field of view for fluorescence lifetime imaging microscopy (FLIM), we also recorded a 2-channel fluorescence-intensity image corresponding to the Hoechst 33342 and enhanced GFP emissions to identify the ruptured micronuclei. The four subregions considered (heterochromatin, euchromatin and intact and ruptured micronuclei) were determined manually according to the following criteria. For heterochromatin, in the Hoechst 33342 channel, bright spots in the nucleus correspond to heterochromatin-enriched regions in 4T1 or MDA-MB-231 cells. For euchromatin, in the Hoechst 33342 channel, regions in the nucleus with low Hoechst 33342 signal were classified as euchromatin-enriched regions. For micronuclei, in the Hoechst 33342 channel, micronuclei were identified as nucleic-acid-positive compartments that were smaller than the main nucleus. They were further classified as intact, if there was no signal in the eGFP channel, or ruptured, if there was a positive signal in the eGFP channel. The subregions selected above were used to segment the lifetime image, and a single lifetime value was obtained as the median value of the entire subregion. For every cell, one lifetime value for heterochromatin, euchromatin and micronuclei (either ruptured or intact) was obtained, for a total of 73 cells (31 intact, 42 ruptured micronuclei) over five biological replicates. Outliers were removed by the MATLAB function ‘isoutlier’. Given that FLIM was performed on live cells we postulate that, as well as chromatin compaction states, it would enable the observation of digested DNA that would otherwise be lost during permeabilization used in the other methods (ATAC-see and ATAC-seq). To address this, we repeated all experiments in *TREX1*-knockout cells as shown in Fig. 3b.

### ATAC-see with immunofluorescence microscopy

Tn5 production, transposome assembly and ATAC-see were performed as described^28^ with some adjustments for subsequent immunofluorescence experiments. In brief, cells were grown on coverslip until 80–90% confluent, and were fixed using 1% paraformaldehyde in PBS at room temperature for 10 min. Permeabilization was done in lysis buffer (10 mM Tris-Cl, pH 7.4, 10 mM NaCl, 3 mM MgCl2, 0.01% Igepal CA-630) for 10 min at room temperature. Coverslips with cells were washed twice with PBS for 5 min each at room temperature. The transposase mixture solution (25 μl of 2× TD buffer, final concentration of 100 nM Tn5-ATTO-59ON and dH2O up to 50 μl) was added on the coverslip and incubated for 30 min at 37 °C in a humid chamber box. Coverslips were then washed three times with PBS containing 0.01% SDS and 50 mM EDTA for 15 min at 55 °C each time. After washing, coverslips were subjected to the immunofluorescence procedure described above, starting from washing with TBS-BSA (1%).

### Super-resolution immunofluorescence microscopy

Images were acquired with an inverted Zeiss LSM880 (Carl Zeiss Microscopy) equipped with an Airy Scan detector (gain 850, digital gain 1) in super-resolution mode. The 848 × 848 pixels images were acquired with a Plan-Apochromat 63×/1.4 NA oil objective using a pixel size of 40 nm and a dwell time of 4.96 μs. The optical configuration used split the laser lines (405 nm at 1% power, 488 nm at 3% and 561 nm at 7%) with a 488/561/633 MBS and an invisible light beam splitter at 405. For the detection we applied a bandpass filter at 495–550 nm and a longpass filter at 570 nm. Each image is acquired as a *z* stack of 5 stacks with an interval of 500 nm; a maximum-intensity projection and Airy Scan processing with default parameters were performed for all images before quantification using the Zeiss ZEN black software. For the ATAC-see images, only two laser lines were used, 405 nm and 594 nm, at 1% and 30% power, respectively, using the optical configuration described above. The number of micronuclei number was scored in 10 different sections per coverslip (3 coverslips accounting for 3 biological replicates) at a magnification of 63×. Missegregation scoring was done to at least 50 cells undergoing anaphase per biological replicate (*n* = 3).

### Purification of micronuclei

Micronucleus purification was performed as described previously^41^, except that the primary nucleus fraction was also collected. In brief, cells were expanded in 245 × 245 × 25 mm dishes and treated with reversine as specified above. Cells were then collected and washed in DMEM. Washed cells were resuspended in prewarmed (37 °C) DMEM supplemented with 10 μg ml^−1^ cytochalasin B (Sigma-Aldrich) at a concentration of around 5 × 10^6^ cells per ml DMEM and incubated at 37 °C for at least 30 min. Subsequently, cells were centrifuged at 300*g* for 5 min and resuspended in cold lysis buffer (10 mM Tris-HCl, 2 mM Mg-acetate, 3 mM CaCl_2_, 0.32 M sucrose, 0.1 mM EDTA, 0.1% (v/v) NP-40, pH 8.5) at a concentration of 10^7^ cells per ml lysis buffer, which was freshly complemented with 1 mM dithiothreitol, 0.15 mM spermine, 0.75 mM spermidine, 10 μg ml^−1^ cytochalasin B and protease inhibitors. Resuspended cells were then dounce-homogenized 10 times using a loose-fitting pestle. Then, lysates were mixed with an equal volume of cold 1.8 M sucrose buffer (10 mM Tris-HCl, 1.8 M sucrose, 5 mM Mg-acetate, 0.1 mM EDTA, pH 8.0) freshly complemented with 1 mM dithiothreitol, 0.3% BSA, 0.15 mM spermine and 0.75 mM spermidine before use. In a 50-ml conical tube, 10 ml of the mixture was then layered on top of a two-layer sucrose gradient (20 ml of 1.8 M sucrose buffer on top of 15 ml 1.6 M sucrose buffer). This mixture was then centrifuged in a swing bucket centrifuge at 950*g* for 20 min at 4 °C. The first resulting 2-ml top fraction is discarded; the next 5–6 ml, mostly containing micronuclei, was collected, and the subsequent 3 ml, mostly containing primary nuclei, was collected in a separate container. Collected fractions were diluted 1:5 with FACS buffer (ice-cold PBS freshly supplemented with 0.3% BSA, 0.1% NP-40 and protease inhibitors). Diluted micronuclei were then centrifuged at 950*g* in a JS-5.2 swing-bucket centrifuge for 20 min at 4 °C. The resulting supernatant was removed by aspiration and either micronuclei or primary nuclei were resuspended in 2–4 ml FACS buffer supplemented with 2 μg ml^−1^ DAPI (but no DAPI was used for micronuclei purification for ATAC-seq and CUT&RUN experiments). Resuspended samples were filtered through a 40-μm ministrainer (PluriSelect) into FACS tubes. Micronuclei were then sorted by FACSAria (BD Biosciences) into FACS buffer at the MSKCC Flow Cytometry Core Facility. Default FSC and DAPI thresholds were lowered, and a log scale was used to visualize the micronuclei. For ATAC-seq micronuclei purification, FSC and mCherry were used instead of FSC and DAPI. Sorted micronuclei were centrifuged at 4,000*g* in a swing-bucket rotor for 20 min at 4 °C and the pellets were stored at −80 °C for later use or processed immediately or ATAC-seq or CUT&RUN experiments.

### DNA extraction

Viably frozen cells were thawed, pelleted and incubated for at least 30 min in 360 μl buffer ATL + 40 μl proteinase K at 55 °C. DNA isolation proceeded with the DNeasy Blood & Tissue Kit (Qiagen) according to the manufacturer’s protocol modified by replacing AW2 buffer with 80% ethanol. DNA was eluted in 60 µl 0.5× buffer AE heated to 55 °C. For nuclei, DNA from nuclei and micronuclei was isolated with the DNeasy Blood & Tissue Kit (Qiagen) according to the manufacturer’s protocol, modified by replacing AW2 buffer with 80% ethanol. DNA was eluted in 60 µl 0.5 × buffer AE heated to 55 °C.

### WGS

After PicoGreen quantification and quality control by Agilent TapeStation, 97–165 ng genomic or micronuclear DNA was sheared using a LE220-plus focused ultrasonicator (Covaris) and sequencing libraries were prepared using the KAPA Hyper Prep Kit (Kapa Biosystems) with modifications. In brief, libraries were subjected to a 0.5 × size selection using aMPure XP beads (Beckman Coulter) after post-ligation clean-up. Libraries were amplified with 5 cycles of PCR and pooled at equimolar ratios. Samples were run on a NovaSeq 6000 in a PE150 run, using the NovaSeq 6000 S4 Reagent Kit (300 cycles) (Illumina). The average coverage per sample was 18×. For micronuclei samples, copy-number analysis was done using HMMcopy^42^ v.1.38 with the MoCaSeq pipeline^43^, setting C57BL-6NJ 6NJ^44^ as the normal pair for all three. Plots for tumour/normal relative copy number ratios (Extended Data Fig. 6g) were also made using MoCaSeq, using its CNV_PlotHMMcopy R script. For the copy-number comparisons with ATAC-seq counts of micronucleus and primary nucleus samples (Extended Data Fig. 6i), reads were mapped to the mm10 genome using BWA-MEM (default settings), and duplicate marking and sorting were done using NovoSort MarkDuplicates (v. 3.08.02), a multi-threaded bam sort/merge tool by Novocraft Technologies. Counts were obtained for 5-kb bins using bedtools (v. 2.25.0) for ATAC-seq and WGS mapped reads. For Fig. 3f, hierarchical clustering was done using the pheatmap package in R. Copy-number profiles were projected onto the clusters by intersecting ATAC-seq peaks with the surrounding 1-kb window of the WGS data. For Extended Data Fig. 6j, copy-number box plots were created by cutting the row dendrogram of the ATAC-seq heat map to produce micronuclei up and down groups and then plotting the corresponding 1-kb windows from the WGS copy-number ratios. For RPE-1 samples, the copy-number variation (CNV; GATK4) pipeline from Basepair was used to determine the copy number.

### CUT&RUN of cell lines

CUTANA CUT&RUN was performed with RPE-1 and DLD-1 samples on an automated protocol (autoCUT&RUN) derived from those previously described^45,46^. In brief, for each CUT&RUN reaction, 500,000 cells (5 million cells per ml prepared in 20 mM HEPES, pH 7.5, 150 mM NaCl, 0.5 mM spermidine, 1 mM trichostatin A, 1× EDTA/EGTA-free complete protease inhibitor) were dispensed to individual wells of a 96-well plate, immobilized onto concanavalin-A beads (EpiCypher) and incubated overnight (4 °C) with 0.5 µg antibody (IgG, H3K4me3, H3K27me3, H3K27ac) (all antibodies validated to histone PTM-defined SNAP-ChIP nucleosome standards). pAG-micronuclease (EpiCypher) was added and activated (2 h at 4 °C), and CUT&RUN-enriched DNA was purified using Serapure beads after mixing at a 2:1 (bead:DNA) ratio. Recovered DNA was quantified using PicoGreen and reactions were normalized to 5 ng DNA (or the entirety of the reaction if less than 5 ng DNA was recovered) before preparing sequencing libraries (CUTANA CUT&RUN Library Prep kit; EpiCypher). All CUT&RUN steps were optimized and performed on Tecan Freedom EVO robotics platforms with gentle rocking for incubation steps and magnetic capture for medium exchange and washing steps as described previously^45,46^.

### CUT&RUN of micronuclei and primary nuclei

CUTANA CUT&RUN was performed with isolated micronuclei and primary nuclei samples using a CUTANA ChIC/CUT&RUN kit (EpiCypher, 14-1048), following the manufacturer’s manual for nuclei samples. Around 50,000 primary nuclei, 1,000,000 intact micronuclei and 100,000 ruptured micronuclei were subjected to CUT&RUN for each replicate. In brief, nuclei were immobilized onto concanavalin-A beads from the kit and incubated overnight (4 °C) with 0.5 µg of antibody (IgG, H3K4me3). pAG-micronuclease (EpiCypher) was added and activated (2 h at 4 °C), and CUT&RUN-enriched DNA was purified using the kit’s purification protocol and reagents. The reaction was used to prepare sequencing libraries using the protocol from the CUTANA CUT&RUN Library Prep kit (EpiCypher).

### RNA-seq

RNA was extracted using the manufacturer’s protocols in the Qiagen RNeasy mini plus kit (Qiagen). Library preparations are TruSeq stranded and sequenced using the poly(A) RNA-sequencing workflow, with a read depth of 80–100 million, 100-base-pair (bp) paired-end reads. Library preparation and sequencing were performed in MSKCC Integrated Genomics Core. RNA-seq data were analysed with a Basepair toolkit using the expression count (STAR) workflow to obtain values of fragments per kilobase of transcript per million mapped reads. Principal component analysis was done using randomized principal component analysis applied to the normalized count matrix of all genes (Extended Data Fig. 8g) or a list of oncogenes and tumour-suppressor genes (Extended Data Fig. 9c) using Partek Flow software, version 10.0 (Partek). Principal component analysis of *TP53*-knockout hTERT RPE-1 cells (Extended Data Fig. 9c) was done using the prcomp function in the stats package (v. 4.2.1) in R (v.4.2.1) and RStudio (version 2022.07.0+548). A GSEA was performed on normalized gene counts from control and lamin-B2-overexpressing *TP53*-knockout hTERT RPE-1 cells treated with reversine or DMSO (Extended Data Fig. 9a,b) using the GSEA software version 4.2.3 (The Broad Institute), with the following parameters: metric for ranking genes, Signal2Noise; weighted enrichment statistics; permutation type, gene set. The gene sets used for GSEA were derived from the ATAC-seq analysis of chromatin accessibility in intact or ruptured micronuclei in 4T1 (Fig. 3f). Mouse gene symbols were converted to human gene symbols using the Ensembl biomaRt package (version 2.48.3). Expression datasets from the TCGA breast-cancer cohort were accessed through cbioportal^47^ (https://www.cbioportal.org/study/summary?id=brca_tcga_pan_can_atlas_2018). GSEA comparing tumours belonging to the top or bottom tertile of the fraction of genome altered, as reported by the TCGA, was performed as described above, and using the same parameters as above (Fig. 3f). The log_2_-transformed fold change was computed using the DESeq2 package (v. 1.36.0), and KEGG pathways (Extended Data Fig. 9d) were generated using the pathview package (version 1.36.0) in Bioconductor (v. 3.15).

### ATAC-seq

The ATAC-seq procedure on cells was done as described previously^48^. The ATAC-seq procedure on nuclei samples was done immediately after isolation using the same protocol but without the lysis step. The ratio of primary nuclei to micronuclei was 1:20. Libraries were sequenced with 50-bp paired-end reads to approximately 50 million reads per sample for cells, whereas for nuclei samples, sequencing was done with 50-bp paired-end reads to 350 million total reads for a triplicate of each sample at the Memorial Sloan Kettering Cancer Center Integrated Genomics Core. For chromosome-4-missegregation ATAC-seq experiments in DLD-1 and RPE-1 cells, the raw fastq data were analysed using the Basepair toolkit. The raw reads were trimmed using fastp to remove low-quality bases from reads (quality <20) and adapter sequences. The trimmed reads were aligned using Bowtie2 as described^49^ to the UCSC genome assembly (hg38). For long-term dnMCAK and micronuclei ATAC-seq experiments in RPE-1 and 4T1 cells, reads were mapped to the hg38 (RPE-1) or mm10 (4T1) genome assembly using Bowtie2 with the following parameters: -X2000 --no-mixed --no-discordant and reads were filtered using samtools for the 1804 FLAG and mapq score of 30. PCR duplicates were identified using the MarkDuplicates function from Picard tools. Peaks were called per sample using macs2 with the following parameters: callpeak -f BAM -g hs --nomodel --shift 37 --extsize 73 --keep-dup all -B --SPMR --call-summits -q 1e-2. Final peaks across each dataset were determined based on an extension of 250 bp from the summit of each peak and merged across all samples using bedtools. Normalized bigwig files were generated using the bamCoverage function from deeptools using reads per kilobase of transcript per million mapped reads normalization. Heat maps for genomic regions were generated using the deeptools computeMatrix scale-region and plotHeatmap functions. Differential peak calling was performed using DESeq2 on raw counts for the merged peaks (an adjusted *P*-value cut-off of 0.01 and log_2_-transformed fold change of 1.5 was applied to the mouse 4T1 cell lines and an adjusted *P*-value of 0.05 and log_2_-transformed fold change of 0.75 was applied to the RPE-1 cell line). The normalized counts for peaks were used to plot the heat maps of differential peaks. Pie charts were generated using the plotAnnoPie function in the ChIPseeker R package. Pathway enrichment was performed on genes associated with differential peaks using the enrichGO function in the Bioconductor package clusterProfiler and similar pathways were merged in R using the ‘simplify’ function with a similarity cut-off of 0.7.

### Micronuclei ATAC-seq and H3K4me3 CUT&RUN analyses

The *k*-means clustering of ATAC and H3K4me3 CUT&RUN data was performed on normalized count matrices with increasing *k* until the clusters became redundant. Hierarchical clustering was done using the pheatmap package in R. Peak annotation pie charts were created in R with the annotatePeak and plotAnnoPie functions from the package ChIPseeker. High-, medium- and low-expression groups were based on normalized expression from RNA-seq results in 4T1 dnMCAK cells and divided using the top, middle and bottom third of TPM (transcript per million) values. Peaks were annotated by gene and assigned to the expression group of the gene. ATAC and H3K4me3 CUT&RUN pie charts were based on differential peak overlaps obtained using bedtools intersect. Scatterplots were created using the normalized ATAC or H4K4me3 signal at ±500 bp surrounding a TSS and plotting the log_2_-transformed fold change of the averaged micronucleus/primary nucleus signal against the normalized gene expression of the matching gene.

### ATAC-seq and CUT&RUN normalization for DLD-1 cells

#### CNV calling from WGS data

WGS CRAM files were obtained from EGA (dataset ID EGAD00001004163) and unmapped from GRCh37 using samtools (version 1.3.1), and then aligned to GRCh38 using BWA-MEM (version 0.7.17-r1188). Copy-number profiles were called using Python software using the CNVKit library (version 0.9.7). Integer copy numbers were obtained using the CNVKit ‘call’ command, and non-integer copy numbers were obtained from the log_2_-transformed copy-number ratios reported in the CNVKit .cns files j.

#### Data filtering

ATAC-seq and CUT&RUN alignments were filtered from the BAM files to remove PCR duplicates, reads aligning with mitochondrial DNA, and alignments with low mapping quality (MAPQ score <15)

#### Signal calculation

We used the ATAC-seq signal not to identify peaks corresponding to active transcriptional sites but to measure the total accessibility of DNA within a region. We generated ATAC-seq read counts for 10,000-bp genomic windows using samtools (version 1.13). We then calculated the signal values for each window by normalizing the read counts by copy number, chromosome length and total ATAC-seq reads across all chromosomes (Supplementary Fig. 5). Windows overlapping with unmappable regions (using the AmpliconSuite-pipeline genome annotation files), or with a copy number close to 0 (<0.3), were excluded from further analyses. The signal calculation for CUT&RUN data was done in the same way. The normalized signal of a clone *c* in a given window *i* with total (ATAC-seq or CUT&RUN) reads *T*(*i*) and copy number *C(i*) was calculated as

$$S\left(c,i\right)=\frac{T\left(i\right)}{C\left(i\right)}\frac{L}{R},$$

where *R* is the total number of ATAC-seq reads mapped across all chromosomes in clone *c*; *L* denotes the sum of ‘effective lengths’ of all the chromosomes. The effective length *l* of a chromosome is calculated as follows. Let *P* denote the expected ploidy (*P* = 1 for the Y chromosome; *P* = 2 otherwise) of the chromosome, and *l(i)*,*C*(*i)* denote the length (in bp) and average copy number, respectively, of window *i* in the chromosome. Then,

$$l=\frac{{\sum }_{i}l\left(i\right)C\left(i\right)}{P}$$

In the case of the Y chromosome, we used only the region up to 26.68 Mbp for the above calculations because the Y chromosome did not have any confidently aligned ATAC reads beyond 26.68 Mbp until the telomeric region in any of the clones (including the parental clone).

### Differential analysis of Y-chromosome accessibility

The normalized ATAC signals of a clone can be compared to the parental signals by taking the log-transformed signal fold changes with respect to the parental signal in the same window.

The signal fold change (*F*) of a clone *c* in a given window *I* is given by:

$$F\left(c,i\right)=\frac{S\left(c,i\right)}{S\left(p,i\right)}$$

where *p* is the parental clone.

### Intra-clone variability and change in accessibility

We computed the mean and variance of log-transformed signal fold changes for each chromosome in a single clone to understand the global changes in accessibility (Supplementary Fig. 5). To control for high fold changes caused by error in windows with low read counts, windows in which neither the clone nor the parent has more than 200 reads were excluded from this calculation.

### Inter-clone variability

To understand whether the Y-chromosome accessibility changes were similar for different clones, we calculated the variance of log-transformed signal fold changes of different clones in the same genomic window. Windows where neither the clone nor the parent has more than 200 reads were excluded. Moreover, only clones with a copy number >0.3 in the window were included for the variance calculation.

### Structural variations and change in accessibility

We used manta (version 1.6.0) to call somatic variants in all the clones using a tumour/normal approach with the parental clone as a normal sample. We then checked for any correlation between the density of variant breakends and the variance of log-transformed signal fold changes. We also compared the signal fold change variance in just the regions containing breakends with the variance across all regions to determine whether the change in accessibility is limited to regions with structural variations.

### Immunofluorescence staining of human samples

The immunofluorescence detection of cGAS, H3K27me3 and H3K27ac were performed at the Molecular Cytology Core Facility of MSKCC using the Discovery Ultra processor (Ventana Medical Systems, Roche). After 32 min of heat (95 °C) and treatment with Cell Conditioning 1 solution (Ventana), the tissue sections were blocked first for 30 min in Background Blocking reagent (Innovex). A mouse monoclonal cGAS antibody (LSBio) was used at a dilution of 1:200. The primary antibody was incubated for 5 h, after which the biotinylated anti-mouse secondary (Vector Labs) was incubated at a concentration of 5.75 µg ml^−1^. Blocker D, streptavidin–HRP and TSA Alexa 488 (Life Tech) were diluted according to the manufacturer’s instructions at 1:150 and incubated for 16 min. A mouse monoclonal antibody for H3K27me3 (Active Motif) was used at a dilution of 1:500. The primary antibody was incubated for 6 h, after which the biotinylated anti-mouse secondary (Vector Labs) was incubated at a concentration of 5.75 µg ml^−1^. Blocker D, streptavidin–HRP and Tyramide-CF594 (Biotium) were diluted according to the manufacturer’s instructions at 1:2,000 for 16 min. A rabbit polyclonal H3K27ac antibody (Abcam) was used at a concentration of 0.05 µg  ml^−1^. The primary antibody was incubated for 6 h, after which the biotinylated goat anti-rabbit IgG (Vector labs) was incubated for 60 min at a concentration of 5.75 µg  ml^−1^. Blocker D, streptavidin–HRP and TSA Alexa 647 (Life Tech) were diluted according to the manufacturer’s instructions at 1:150 and incubated for 16 min. All slides were counterstained in 5 µg  ml^−1^ DAPI (Sigma D9542) for 5 min at room temperature, mounted with anti-fade mounting medium Mowiol [Mowiol 4–88 (CALBIOCHEM)] and a coverslip was added. The 956 × 956 pixels *z* stack images were acquired with an inverted Zeiss LSM880, using the optical configuration described above. The acquisition was performed with a pixel size and dwell time of 40 nm and 4.39 μs, respectively, and using the following lasers and relative powers: 405 nm at 0.4%, 488 nm at 3%, 561 nm at 0.5% and 647 nm at 0.5%. The images were processed as described above, using the Zeiss ZEN black software.

### 5-ethynyluridine-marked transcript detection and immunofluorescence

To detect nascent transcripts, cells were incubated for 30 min with 1 mM 5-ethynyluridine. Incorporation of 5-ethynyluridine was detected using the Click-iT RNA Alexa Fluor 594 Imaging Kit according to the manufacturer’s instructions (ThermoFisher). Samples were then incubated overnight in antibody. Sample staining and quantification was then performed using the immunofluorescence microscopy and histone PTM quantification methods described above with the following changes: paired measurements were performed for each micronucleus and its primary nucleus. For all measurements a *z*-score was calculated and experimental replicates were combined. Samples with a positive *z*-score were defined as having H3K27ac-positive staining.

### FISH immunofluorescence

For immunofluorescence combined with DNA FISH, the immunofluorescence procedure was performed as described above followed by fixation in 1% paraformaldehyde for 15 min and subsequently in Carnoy’s fixative at −20 °C for 15 min. Chromosome paint probes were purchased from MetaSystems. To perform FISH on interphase cells, unsynchronized cells cultured in chamber slides were washed with PBS and fixed with Carnoy’s fixative for 15 min at room temperature. The slides were then dehydrated in sequential washes in 70% ethanol, 85% ethanol and 100% ethanol and then air-dried for 2 min. FISH probes were applied to interphase cells on glass slides and sealed with a coverslip. Samples and probes were co-denatured at 75 °C for 2 min, followed by sealing with rubber cement and overnight hybridization at 37 °C in a humidified chamber. Slides were washed in 2× SSC three times for 30 s. Slides were rinsed in PBS, stained with DAPI, dehydrated in sequential washes in 70% ethanol, 85% ethanol and 100% ethanol and mounted in ProLong Gold anti-fade mounting solution.

### Live cell imaging

On the day before imaging, 2,500 cells were plated in each well of an Ibidi 8-well plate chamber (µ-Slide 8 Well high Glass Bottom, Ibidi). After 24 h, cells were washed twice with PBS and stained with NucSpot Live 488 (Biotium) or with NucRed Live 647 (Invitrogen) dyes, following the manufacturer’s instructions. When using tNucSpot Live 488, the dye was incubated with the cells in the imaging solution (Live Cell Imaging Solution (Invitrogen) supplemented with 10% FBS). In the case of NucRed Live 647, cells were washed 4 times with Live Cell Imaging Solution and then imaged in Live Cell Imaging Solution supplemented with 10% FBS. Cells were imaged on an Axio Observer Zeiss wide-field microscope equipped with a Plan-Apochromat 20x/0.8 M27 air objective, incubation chamber with controlled temperature, CO_2_ and humidity, and a Hamamatsu camera. Images were acquired every 10 min in the 488 and 647 channels, with the light source X-Cyte lamp at 20% power for 48 h.

### Chromosome 4 missegregation system

RPE-1 cells were transfected with GFP–Cas9 (IDT) using the target sequence TTTAGTGCCCGGCCGCAAGG using the IDT Alt-R CRISPR–Cas9 sgRNA, 2 nmol platform final sequence: mU*mU*mU* rArGrU rGrCrC rCrGrG rCrCrG rCrArA rGrGrG rUrUrU rUrArG rArGrC rUrArG rArArA rUrArG rCrArA rGrUrU rArArA rArUrA rArGrG rCrUrA rGrUrC rCrGrU rUrArU rCrArA rCrUrU rGrArA rArArA rGrUrG rGrCrA rCrCrG rArGrU rCrGrG rUrGrC mU*mU*mU* rU. We used Lipofectamine 3000 (ThermoFisher) and followed the manufacturer’s protocol. After 48 h, single cells were sorted into individual wells and clones were validated using chromosome 4 paint (Metasystems) according to the manufacturer’s protocol. Clones were screened by DAPI staining to evaluate for bridge formation and micronucleation rate. A subset of clones underwent secondary validation using chromosome 4 paint (Metasystems) according to the manufacturer’s protocol. Validated clones were then processed for DNA-seq, RNA-seq and ATAC-seq as mentioned above.

### Histone extraction and digestion for mass spectrometry

Histone proteins were extracted from the cell pellet as described^50^ to ensure good-quality identification and quantification of single histone marks. In brief, histones were acid-extracted with chilled 0.2 N sulfuric acid (5:1, sulfuric acid:pellet) and incubated with constant rotation for 4 h at 4 °C, followed by precipitation with 33% trichloroacetic acid overnight at 4 °C. The supernatant was then removed and tubes were rinsed with ice-cold acetone containing 0.1% HCl, centrifuged and rinsed again using 100% ice-cold acetone. After the final centrifugation, the supernatant was discarded and the pellet was dried using a vacuum centrifuge. The pellet was next dissolved in 50 mM ammonium bicarbonate, pH 8.0, and histones were subjected to derivatization as described previously^50^. In brief, 5 µl propionic anhydride and 14 µl ammonium hydroxide (all Sigma Aldrich) were added to samples to balance the pH at 8.0. The mixture was incubated for 15 min and the procedure was repeated. Histones were then digested with 1 µg of sequencing-grade trypsin (Promega) diluted in 50 mM ammonium bicarbonate (1:20, enzyme:sample) overnight at room temperature. The derivatization reaction was repeated to derivatize peptide N termini. The samples were finally dried in a vacuum centrifuge. Before mass-spectrometry analysis, samples were desalted using a 96-well plate filter (Orochem) packed with 1 mg Oasis HLB C-18 resin (Waters). The samples were next resuspended in 100 µl of 0.1% trifluoroacetic acid (TFA) and loaded onto the HLB resin, which was previously equilibrated using 100 µl of the same buffer. After washing with 100 µl of 0.1% TFA, the samples were eluted with 70 µl of buffer containing 60% acetonitrile and 0.1% TFA and then dried in a vacuum centrifuge.

### Liquid chromatography tandem mass spectrometry acquisition

Samples were resuspended in 10 µl of 0.1% TFA and loaded onto a Dionex RSLC Ultimate 300 (Thermo Scientific), coupled online with an Orbitrap Fusion Lumos (Thermo Scientific). Chromatographic separation was performed with a two-column system, consisting of a C-18 trap cartridge (300 µm inner dimension, 5 mm length) and a picofrit analytical column (75 µm inner dimension, 25 cm length) packed in-house with reversed-phase Repro-Sil Pur C18-AQ 3 µm resin. Histone peptides were separated using a 30 min gradient from 1–30% buffer B (buffer A, 0.1% formic acid; buffer B, 80% acetonitrile + 0.1% formic acid) at a flow rate of 300 nl min^−1^. The mass spectrometer was set to acquire spectra in a data-independent acquisition mode. Briefly, the full MS scan was set to 300–1,100 *m*/*z* in the orbitrap with a resolution of 120,000 (at 200 *m*/*z*) and an AGC target of 5 × 10^−5^. Tandem mass spectrometry (MS/MS) was performed in the orbitrap with sequential isolation windows of 50 *m*/*z* with an AGC target of 2 × 10^−5^ and a higher-energy C-trap dissociation collision energy of 30. Histone peptide raw files were imported into EpiProfile 2.0 software^51^. From the extracted ion chromatogram, the area under the curve was obtained and used to estimate the abundance of each peptide. To achieve the relative abundance of PTMs, the sum of all different modified forms of a histone peptide was considered as 100% and the peak area of the particular peptide was divided by the total area for that histone peptide in all its modified forms. The relative ratio of two isobaric forms was estimated by averaging the ratio for each fragment ion with different mass between the two species. The resulting peptide lists generated by EpiProfile were exported to Microsoft Excel and further processed for a detailed analysis, as described below. Histone PTM quantification is done automatically using EpiProfile 2.0, a software specifically designed to extract histone peptide signals in mass-spectrometry raw files^51^. The abundance of (un)modified histone peptides is extracted by performing extracted ion chromatography, that is, an area under the curve of signals corresponding to the peptide calculated mass. To convert raw peak area into relative peptide abundance, the sum of the unmodified and all modified forms for a given histone peptide, that is, all forms with the same underlying sequence, is considered as the total. Next, the area of each peptide is divided by this total to obtain a normalized relative abundance in percentage. In the case of isobaric peptides, that is, peptides with the same intact mass (such as H3K18ac and H3K23ac), the MS/MS fragment ions are used first to calculate the ratio between the two species and split the peak area before the percentage calculation. Finally, the total abundance of a given PTM is obtained by summing the relative abundance of the peptides that contain that modification. For instance, the final relative abundance of H3K9me3 (occurring on the peptide amino acids 9–17) is obtained by summing the relative abundances of peptides modified as H3K9me3, K3K9me3K14ac, H3K9me3S10 and H3K9me3S10pK14ac. It is important to note that changes in histone PTMs in micronuclei could not be attributed to differences in the total levels of histone H3 between primary nuclei and micronuclei (LC-MS/MS AUC (liquid chromatography-mass spectrometry/mass spectrometry area under the curve) ± s.e.m. = 3.05  × 10^10^ ± 8.85 × 10^9^ and 3.39 × 10^10^ ± 1.36 × 10^10^ for primary nuclei and micronuclei, respectively, *P* = 0.85, two-sided *t*-test) or specific degradation of the histone tail, as some of the PTMs were differentially enriched on the same lysine residue (for example, loss of acetylation and gain of trimethylation on H3K27).

### Reporting summary

Further information on research design is available in the Nature Portfolio Reporting Summary linked to this article.

## Online content

Any methods, additional references, Nature Portfolio reporting summaries, source data, extended data, supplementary information, acknowledgements, peer review information; details of author contributions and competing interests; and statements of data and code availability are available at 10.1038/s41586-023-06084-7.

## Supplementary information

Supplementary Information This file contains Supplementary Tables 1 and 2, legends for Supplementary Tables 3–6 and Supplementary Figs. 1–7.

Reporting Summary

Supplementary Table 3 List of pathways for which the genes are more accessible in micronuclei than in primary nuclei. Pathway enrichment was determined using an over-representation statistical test (a one-sided version of Fisher’s exact test) and *P* values were adjusted for multiple hypothesis testing using the Benjamini-Hochberg correction.

Supplementary Table 4 List of differentially accessible genes from micronuclei and primary nuclei ATAC-seq on 4T1 cells.

Supplementary Table 5 List of genes from each principal component for the principal component analysis shown in Extended Data Fig. 8f.

Supplementary Table 6 List of hallmark pathways from GSEA analysis on the RNA-seq counts from long-term reversine-treated RPE-1 cells experimental system (Extended Data Fig. 8a).

## Data Availability

The ATAC-seq, RNA-seq and CUT&RUN data generated in this study have been deposited and are publicly available in the NCBI Gene Expression Omnibus database under accession code GSE186589. The WGS data generated in this study have been deposited and are publicly available in the NIH Sequence Read Archive with accession number PRJNA882761. Mass spectrometry raw files are publicly available and deposited in the repository Chorus (https://chorusproject.org/) under project number 1790. The TCGA dataset used was Breast Invasive Carcinoma (TCGA, PanCancer Atlas, https://www.cbioportal.org/study/summary?id=brca_tcga_pan_can_atlas_2018). WGS files from EGA (dataset ID EGAD0000100416) was used in DLD-1 CEN-SELECT samples analysis. Source data are provided with this paper.

## Source data

Source Data Fig. 1
Source Data Fig. 2
Source Data Fig. 3
Source Data Fig. 4
Source Data Fig. 5
Source Data Extended Data Fig. 1
Source Data Extended Data Fig. 2
Source Data Extended Data Fig. 3
Source Data Extended Data Fig. 4
Source Data Extended Data Fig. 5
Source Data Extended Data Fig. 6
Source Data Extended Data Fig. 7
Source Data Extended Data Fig. 8
Source Data Extended Data Fig. 9
Source Data Extended Data Fig. 10

## Extended data

Extended data is available for this paper at 10.1038/s41586-023-06084-7.

## Code availability

From whole-genome sequencing results of DLD-1 cells, copy-number profiles were called using CNVKit (v.0.9.7). Integer copy numbers were obtained using the CNVKit ‘call’ command, and non-integer copy numbers were obtained from the log_2_-transformed CN ratios reported in the CNVKit .cns files using the convert_cns_to_bed.py script of the AmpliconSuite-pipeline (https://github.com/jluebeck/AmpliconSuite-pipeline).
