# Supplementary material for: Epigenetic dysregulation from chromosomal transit in micronuclei
Source: Nature. 2023 Jun 7;619(7968):176–83. doi: 10.1038/s41586-023-06084-7 (PMC10322720; doi:10.1038/s41586-023-06084-7)
Supplement: Supplementary file 1 — This file contains Supplementary Tables 1 and 2, legends for Supplementary Tables 3–6 and Supplementary Figs. 1–7. [file 41586_2023_6084_MOESM1_ESM.pdf]

nature portfolio

https://doi.org/10.1038/s41586-023-06084-7

Supplementary information

Epigenetic dysregulation from chromosomal transit in micronuclei

In the format provided by the authors and unedited

Supplementary Information

Epigenetic dysregulation from chromosomal transit in micronuclei

Albert S. Agustinus[1,2], Duaa Al-Rawi[1,3*], Bhargavi Dameracharla[4*], Ramya Raviram[5*], Bailey S. C. L. Jones[6], Stephanie Stransky[7], Lorenzo Scipioni[8], Jens Luebeck[4], Melody Di Bona[1], Danguole Norkunaite[1], Robert M. Myers[5,9], Mercedes Duran[1], Seongmin Choi[10], Britta Weigelt[11], Shira Yomtoubian[12], Andrew McPherson[10], Eléonore Toufektchan[13], Kristina Keuper[14], Paul S. Mischel[15], Vivek Mittal[121], Sohrab P. Shah[10], John Maciejowski[13], Zuzana Storchova[14], Enrico Gratton[8], Peter Ly[16], Dan Landau[5,17], Mathieu F. Bakhoum[6,18,19], Richard P. Koche[20], Simone Sidoli[7], Vineet Bafna[4], Yael David[2,21,22,#], Samuel F. Bakhoum[1,23,#]

[1] Human Oncology and Pathogenesis Program, Memorial Sloan Kettering Cancer Center, New York, NY
[2] Pharmacology Graduate Program, Weill Cornell Medicine, New York, NY
[3] Department of Medicine, Memorial Sloan Kettering Cancer Center, New York, NY
[4] Department of Computer Science, University of California San Diego, La Jolla, CA
[5] New York Genome Center, New York, NY
[6] Department of Ophthalmology and Visual Science, Yale University School of Medicine, New Haven, CT
[7] Department of Biochemistry, Albert Einstein College of Medicine, New York, NY
[8] School of Engineering, University of California Irvine, Irvine, CA
[9] Tri-institutional MD-PhD Program, New York, NY
[10] Computational Oncology, Department of Epidemiology and Biostatistics, Memorial Sloan Kettering Cancer Center, New York, NY
[11] Department of Pathology and Laboratory Medicine, Memorial Sloan Kettering Cancer Center, New York, NY
[12] Department of Cell and Developmental Biology, Weill Cornell Medicine, New York, NY
[13] Molecular Biology Program, Memorial Sloan Kettering Cancer Center, New York, NY
[14] Department of Molecular Genetics, University of Kaiserslautern, Kaiserslautern, Germany
[15] Department of Pathology, Stanford University, School of Medicine and Stanford ChEM-H, Stanford, CA
[16] Department of Pathology, University of Texas Southwestern Medical Center, Dallas, TX
[17] Meyer Cancer Center, Weill Cornell Medicine, New York, NY
[18] Department of Pathology, Yale University School of Medicine, New Haven, CT
[19] Yale Cancer Center, Yale University, New Haven, CT
[20] Center for epigenetics research, Memorial Sloan Kettering Cancer Center, New York, NY
[21] Chemical Biology Program, Memorial Sloan Kettering Cancer Center, New York, NY
[22] Tri-institutional PhD Program in Chemical Biology, New York, NY
[23] Department of Radiation Oncology, Memorial Sloan Kettering Cancer Center, New York, NY
* Equal contribution

# Corresponding authors:
Yael David, Ph.D.
Chemical Biology Program
Memorial Sloan Kettering Cancer Center
New York, NY, 10065
Email: davidshy@mskcc.org
Phone: 646-888-2127

Samuel F. Bakhoum, M.D., Ph.D.
Human Oncology and Pathogenesis Program
Department of Radiation Oncology
Memorial Sloan Kettering Cancer Center
New York, N.Y., 10065
Email: samuel.bakhoum@gmail.com
Phone: 212-639-5749

Table of Contents

Contents	Page Number
Table of Contents	1
Supplementary Table 1	2
Supplementary Table 2	3
Titles / notes for supplementary tables 3, 4, 5 and 6	3
Supplementary Figure 1	4
Supplementary Figure 2	5
Supplementary Figure 3	6
Supplementary Figure 4	7
Supplementary Figure 5	8
Supplementary Figure 6	9
Supplementary Figure 7	10

Supplementary Table 1: Antibodies used in immunofluorescence

Antibody Against	Company	Catalog Number	Dilution
H3K4Me3	Abcam	ab8580	1/1000
H3K9Me3	Abcam	ab8898	1/500
H3K14Ac	Abcam	52946	1/250
H3K27Me3	Active Motif	61017	1/1000
H3K27Ac	Abcam	ab4729	1/1000
H3K36Me2	Cell Signaling Technology	C75H12	1/200
H3K36Me3	Abcam	ab9050	1/1000
H2AK119Ub	Cell Signaling Technology	8240	1/1600
H2BK120Ub	Cell Signaling Technology	5546S	1/1600
cGAS (human)	LSBio	LS-C757990	1/1000
cGAS (mouse)	Cell Signaling Technology	31659S	1/1000
KDM1A	Abcam	ab17721	1/1000
PHC2	Thermo Fisher Scientific	PA5-61504	1/50
RNF40	Abcam	ab126959	1/250
pRpb1 CTD (ser5)	Cell Signaling Technology	13523S	1/250
Alexa fluor plus 594 donkey anti mouse	Invitrogen	A32744	1/1000
Alexa fluor plus 594 donkey anti rabbit	Invitrogen	A32754	1/1000
Alexa fluor plus 488 donkey anti mouse	Invitrogen	A32766	1/1000
Alexa fluor plus 488 donkey anti rabbit	Invitrogen	A32790	1/1000

Supplementary Table 2: Antibodies used in immunoblotting

Antibody Against	Company	Catalog Number	Dilution
Lamin B2	Abcam	ab151735	1/2000
β-actin	Abcam	ab6276	1/10000
Histone H2A	Invitrogen	MA3-047	1/1000
Histone H2B	Abcam	ab193203	1/1000
Lamin A	Thermo Fisher Scientific	MA1-06101	1/1000
IRDye 680 RD goat anti mouse	LI-COR	926-68070	1/1000
IRDye 680 CW donkey anti rabbit	LI-COR	926-32213	1/1000

Titles / notes for separate supplementary tables

Supplementary Table 3: List of pathways whose genes are more accessible in micronuclei relative to primary nuclei. Pathway enrichment was determined using an over-representation statistical test (a one-sided version of Fisher's exact test) and p-values were adjusted for multiple hypothesis testing via the Benjamini-Hochberg correction.

Supplementary Table 4: List of differentially accessible genes from micronuclei and primary nuclei ATAC-Seq on 4T1 cells.

Supplementary Table 5: List of genes from each principal component on principal component analysis shown in Extended Data Fig. 8f.

Supplementary Table 6: List of hallmarks pathways from GSEA analysis on the RNA-Seq counts from long-term reversine-treated RPE-1 cells experimental system (Extended Data Fig. 8a).

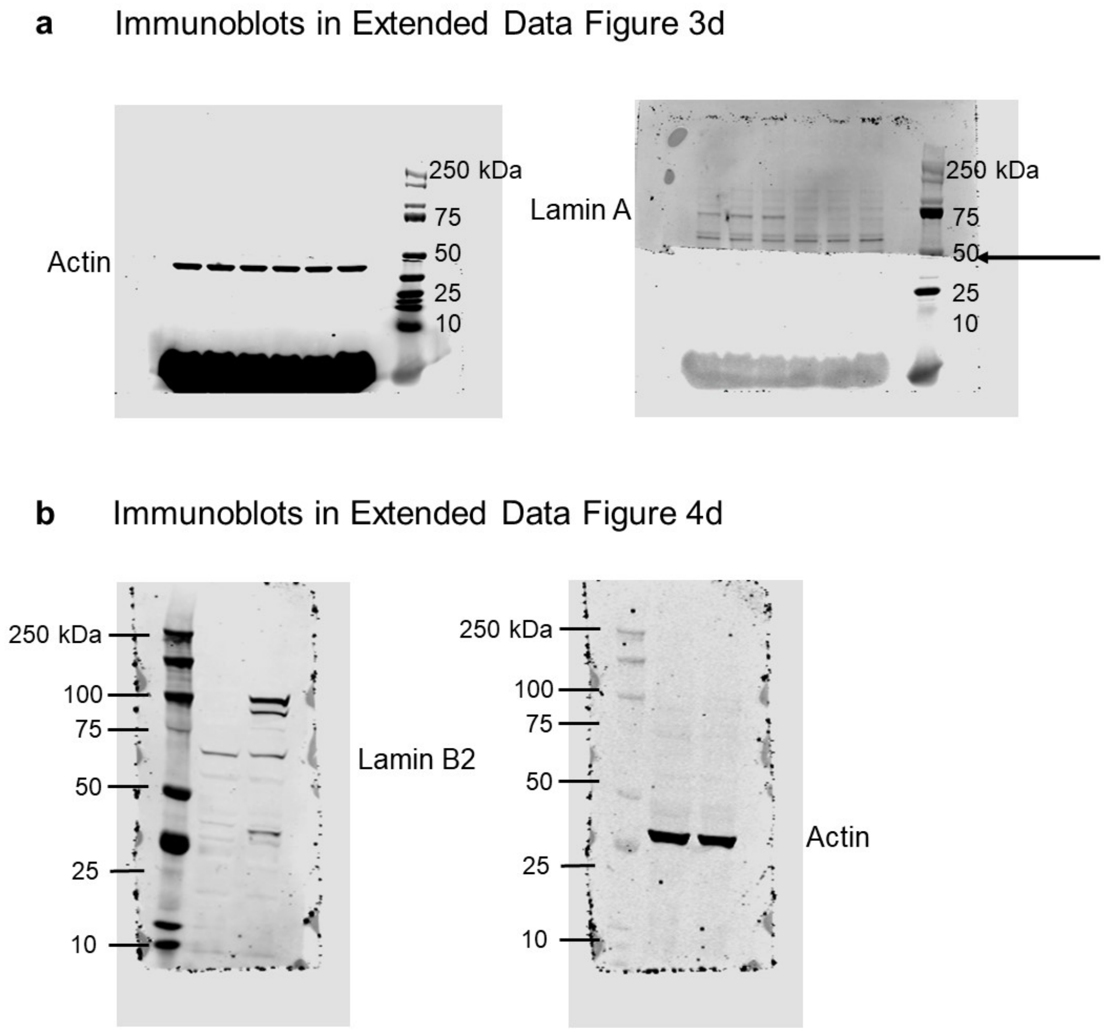

Supplementary Figure 1: a, Full immunoblots from Extended Data Fig. 3d. Membrane was cut at 50 kDa to allow simultaneous staining of Lamin A and Actin (the site where membrane was cut is denoted by arrow). **b,** Full immunoblots from Extended Data Fig. 4d. . Primary antibodies used are denoted beside each corresponding band. Molecular weight markers are in kDa.

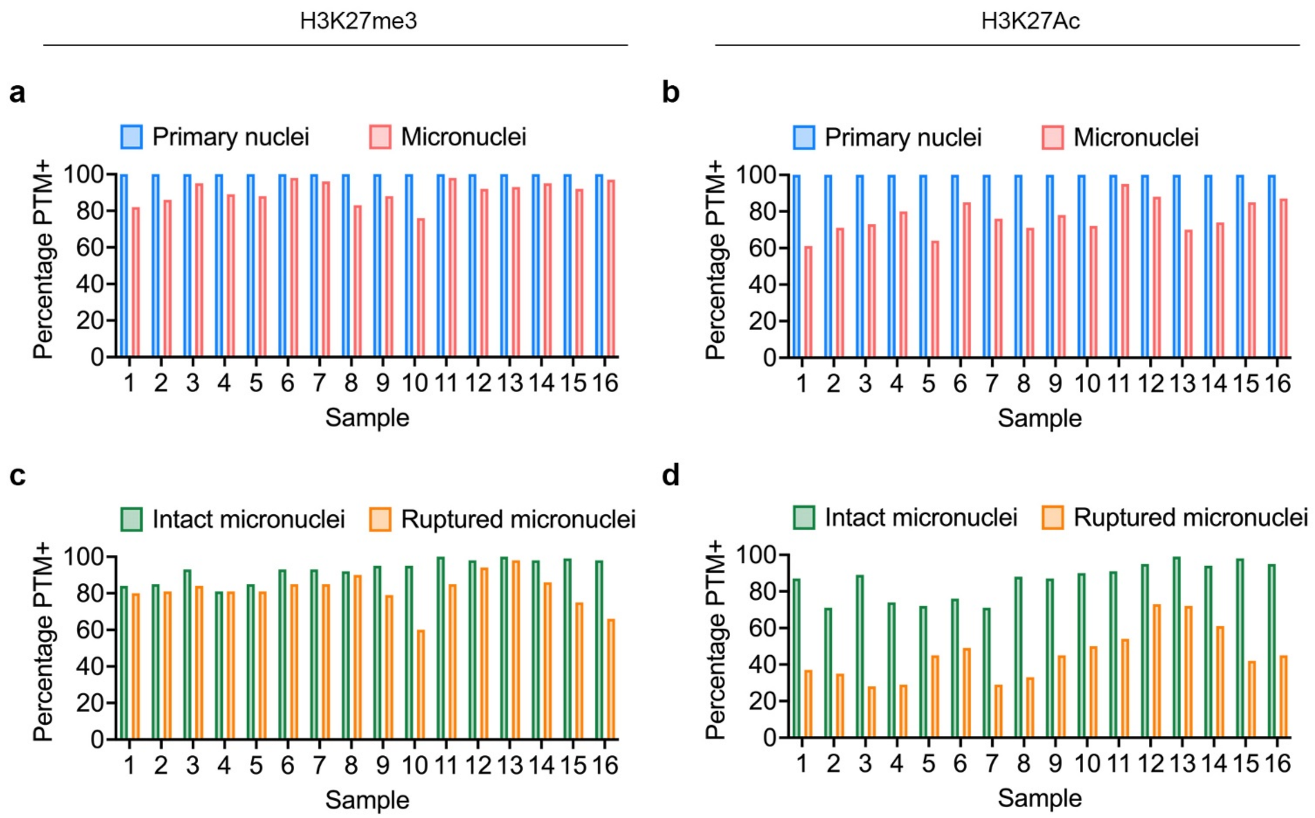

Supplementary Figure 2: **a-b,** The percentage of primary nuclei and micronuclei that are positive for for H3K27me3 (a) or H3K27Ac (b) in individual human HGSOC tumor samples. **c-d,** The percentage of intact (cGAS negative) and ruptured (cGAS positive) micronuclei that are positive for H3K27me3 (c) or H3K27Ac (d) in individual human HGSOC tumor samples.

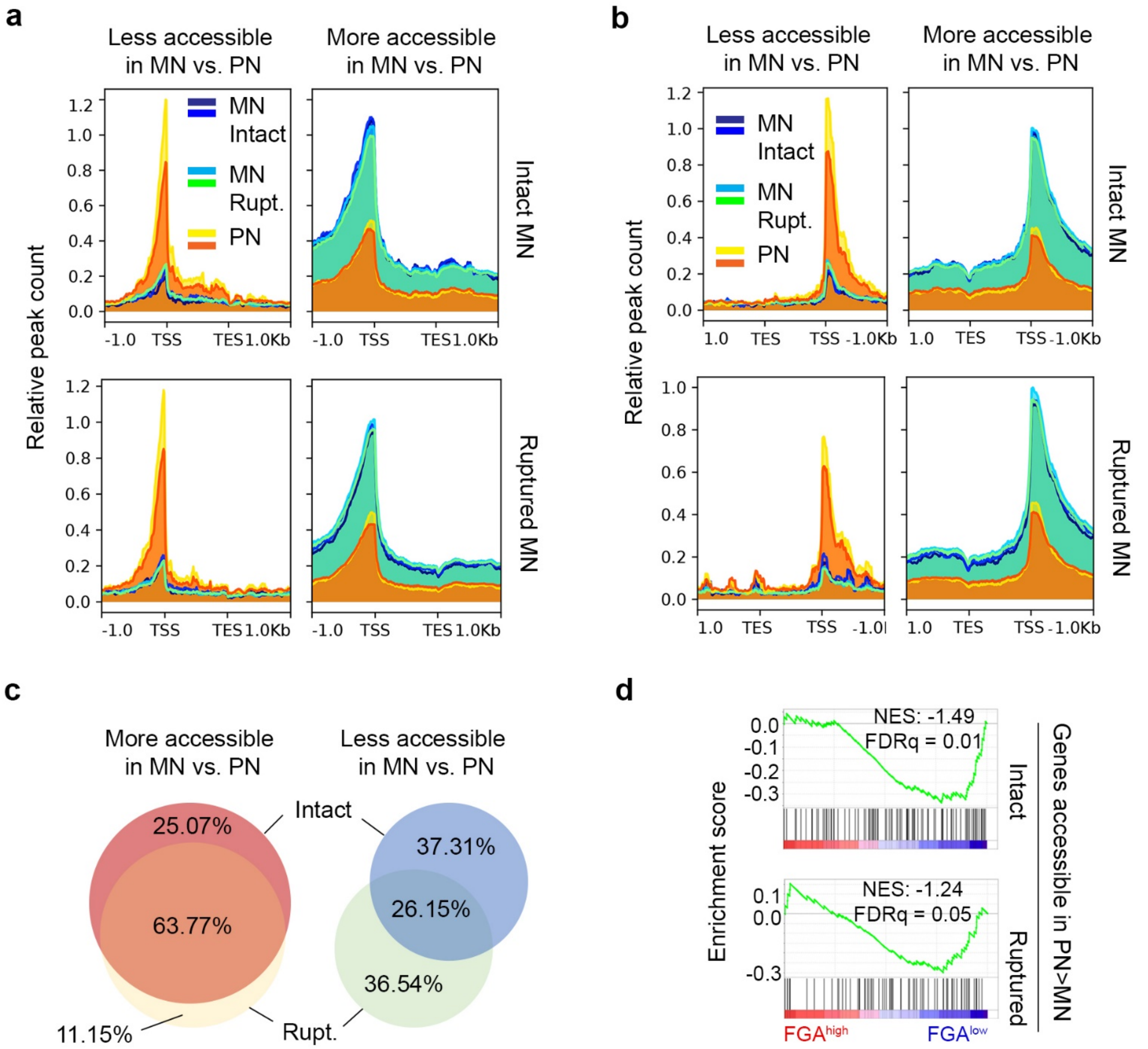

Supplementary Figure 3: **a,** Density plot showing ATAC-seq peak counts of differentially accessible positive strand genes in intact and ruptured micronuclei, from regions that are less accessible in MN (left) or more accessible in MN (right) vs. PN in 4T1 cells. **b,** Density plot showing ATAC-seq peak counts of differentially accessible negative strand genes in intact and ruptured micronuclei, from regions that are less accessible in MN (left) or more accessible in MN (right) vs. PN in 4T1 cells. **c,** Venn diagram representing the overlaps of in promoter accessibilities between intact and ruptured (Rupt.) micronuclei (MN) each relative to primary nuclei (PN) of 4T1 cells. **d, ,** Enrichment plots of genes whose promoters are less accessible in micronuclei compared with primary nuclei in 4T1 cells in comparison to human breast tumors belonging to the top (FGA^{high}) or bottom (FGA^{low}) quartile of fraction genome altered according to TCGA.

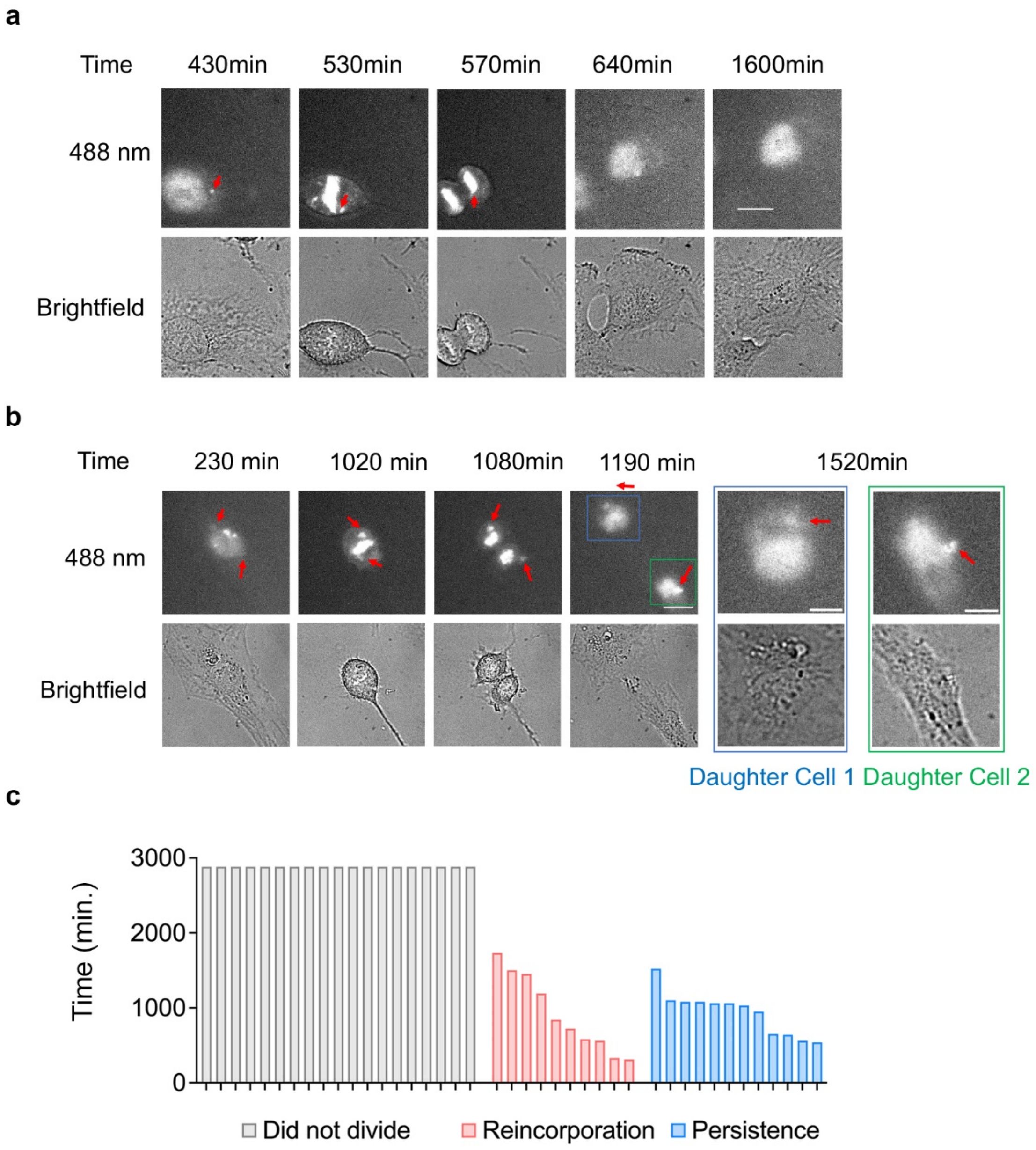

Supplementary Figure 4: **a,** Representative time-lapse imaging of RPE-1 p53 KO cells treated long-term with reversine (P8) showing reincorporation of a micronucleus (10 observations from live cell imaging showing reincorporation in total). DNA is stained using fluorescent live cell imaging dye (488 nm) on the top panels. Brightfield channel is shown in the bottom panels. Arrows point to micronucleus. Scale bar 10 μm. **b,** Representative time-lapse imaging of RPE-1 p53 KO cells treated long-term with reversine (P8) showing persistence of two micronuclei in two daughter cells (boxed in blue and green, 12 observations from live cell imaging showing reincorporation in total) . DNA is stained using fluorescent live cell imaging dye (488 nm) on the top panels. Brightfield channel is shown in the bottom panels. Arrows point to micronuclei. Scale bar 10 μm. **c,** Analysis of micronuclei fate in RPE-1 P53 KO cells treated long-term with reversine (P8) from live-cell imaging experiment for 48 hours (2880 minutes). Value indicates time when corresponding event occurs, either micronuclei continued to exist in cells that did not divide during 48 hours of imaging (gray), micronuclei reincorporated during mitosis (red), or micronuclei persisted after mitosis (blue). All persisting micronuclei remained in cells until the end of live-cell imaging session.

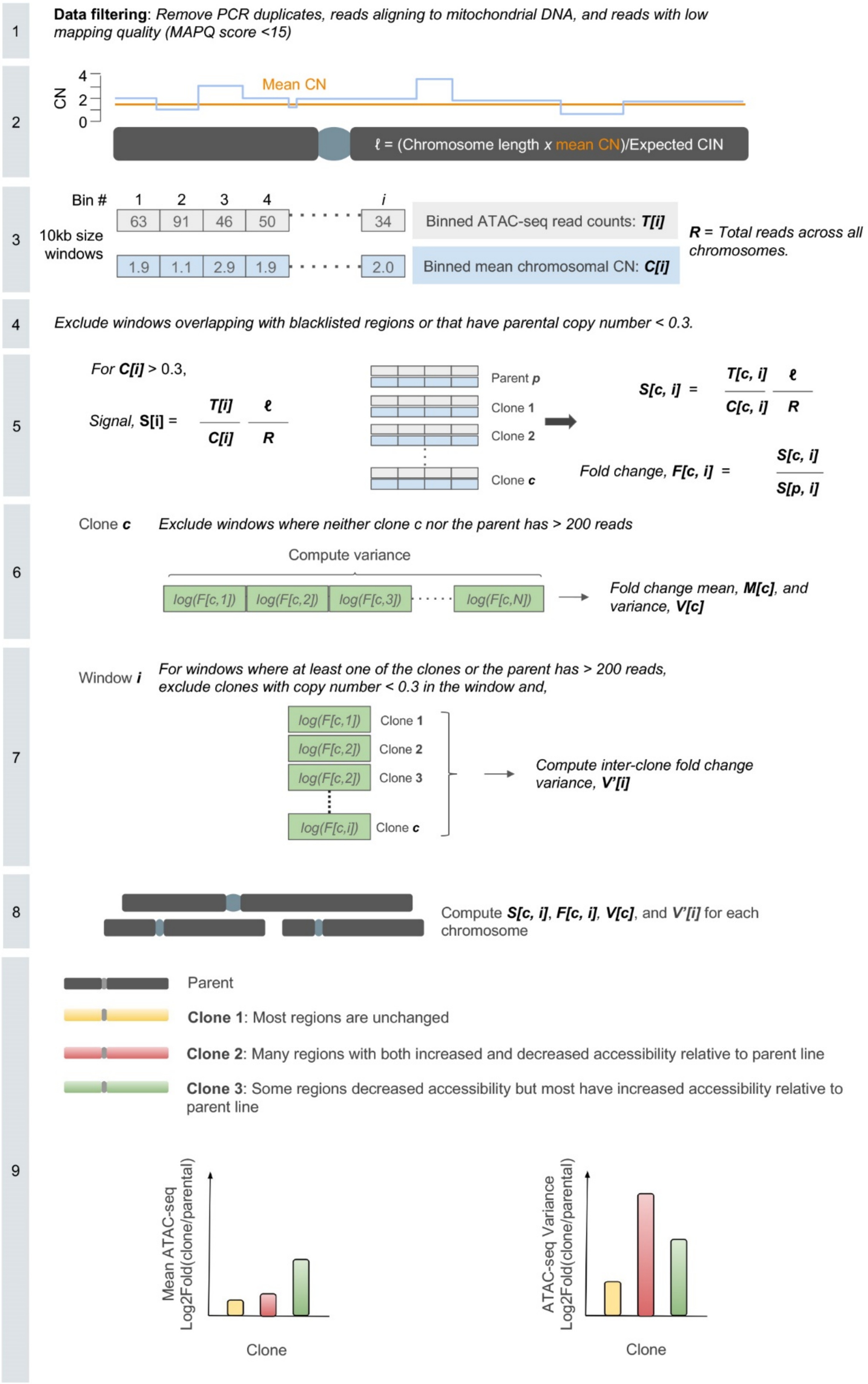

Supplementary Figure 5: Experimental schematic for DLD-1 ATAC-seq peaks normalization to copy number. More details can be found in the methods section "ATAC-seq normalization for DLD-1 cells".

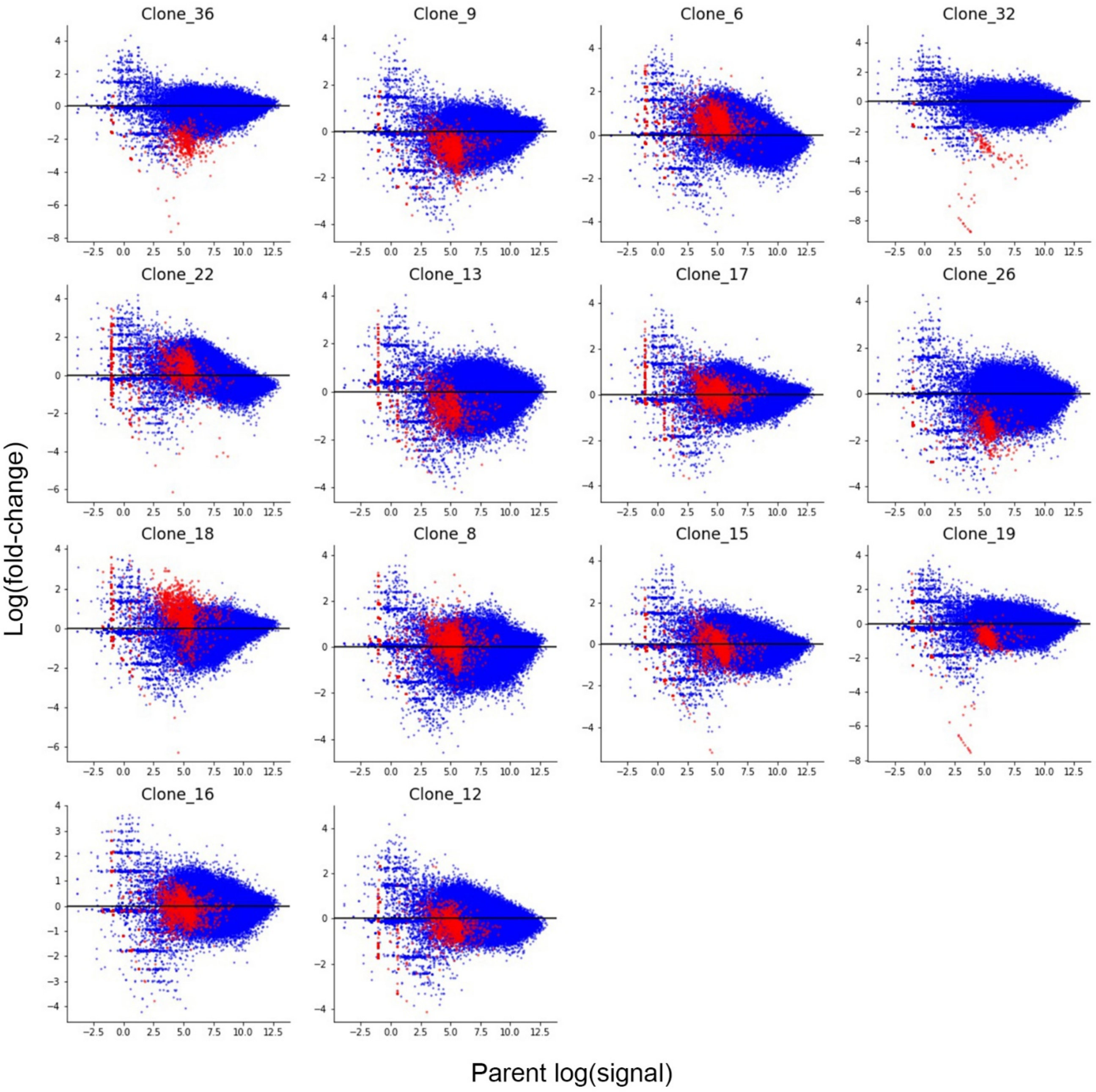

Supplementary Figure 6: Fold change distribution where y-axis represents the value of each clone's fold change of the genomic window compared to the parental cell line, while x-axis represents the value of the parental cells' signal in the same window. Red dots = Y chromosomes, blue dots = autosomes.

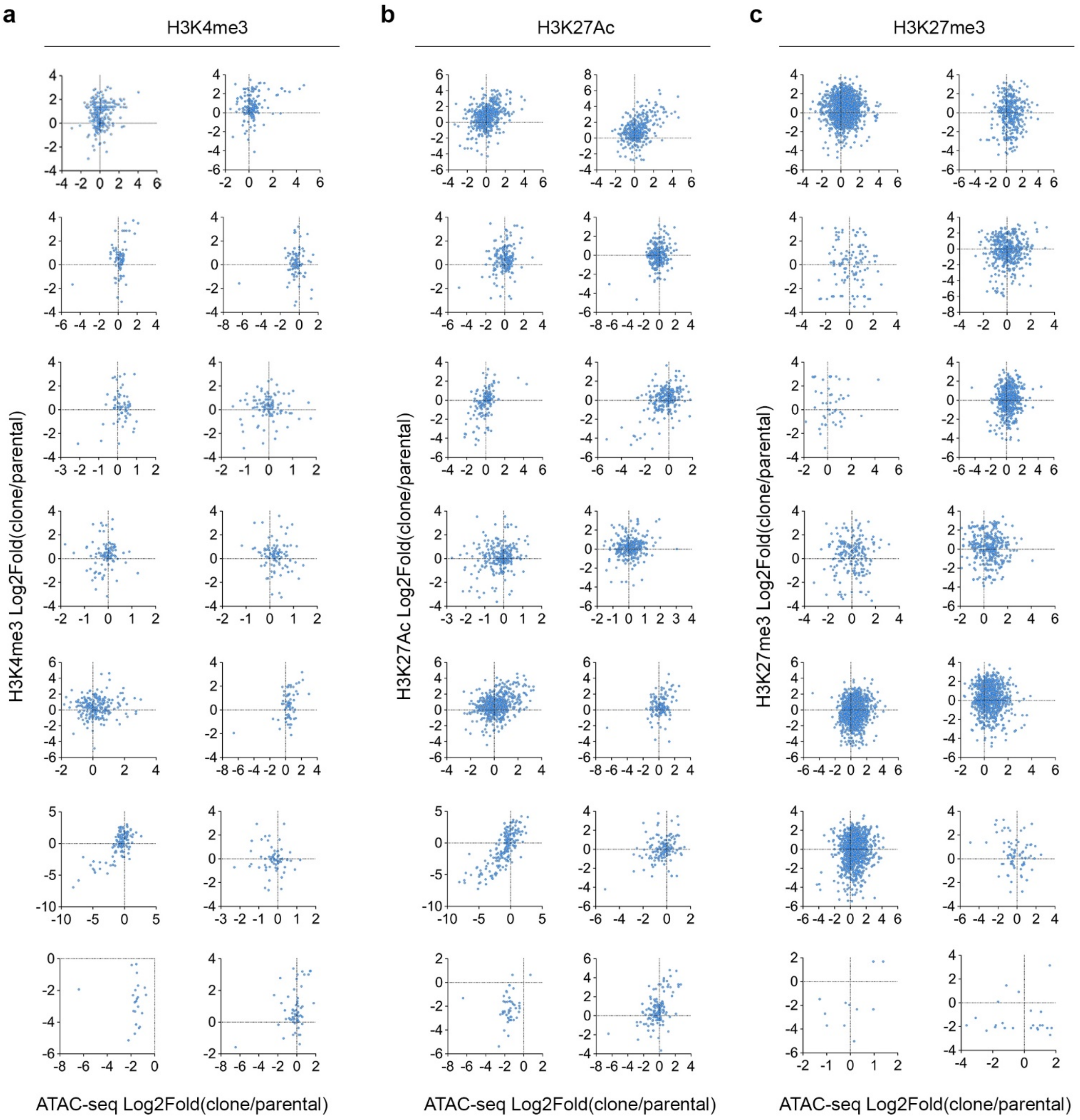

Supplementary Figure 7: a, Scatter plot showing the comparison of the log2 fold change of H3K4me3 CUT&RUN reads (y-axis) vs. ATAC-seq read counts (x-axis) in a given region between all individual DLD-1 CEN-SELECT single cell clones vs. parental. **b,** Scatter plot showing the comparison of the log2 fold change of H3K27Ac CUT&RUN reads (y-axis) vs. ATAC-seq read counts (x-axis) in a given region between all individual DLD-1 CEN-SELECT single cell clones vs. parental. **c,** Scatter plot showing the comparison of the log2 fold change of H3K27me3 CUT&RUN reads (y-axis) vs. ATAC-seq read counts (x-axis) in a given region between all individual DLD-1 CEN-SELECT single cell clones vs. parental.
