# Supplementary material for: Epigenetic dysregulation from chromosomal transit in micronuclei
Source: Nature. 2023 Jun 7;619(7968):176–83. doi: 10.1038/s41586-023-06084-7 (PMC10322720; doi:10.1038/s41586-023-06084-7)
Supplement: Supplementary file 2 — Reporting Summary [file 41586_2023_6084_MOESM2_ESM.pdf]

Corresponding author(s): Samuel Bakhoum

Last updated by author(s): Mar 20, 2023

Reporting Summary

Nature Portfolio wishes to improve the reproducibility of the work that we publish. This form provides structure for consistency and transparency in reporting. For further information on Nature Portfolio policies, see our Editorial Policies and the Editorial Policy Checklist.

Statistics

For all statistical analyses, confirm that the following items are present in the figure legend, table legend, main text, or Methods section.

n/a	Confirmed	
☐	☒	The exact sample size (*n*) for each experimental group/condition, given as a discrete number and unit of measurement
☐	☒	A statement on whether measurements were taken from distinct samples or whether the same sample was measured repeatedly
☐	☒	The statistical test(s) used AND whether they are one- or two-sided<br>*Only common tests should be described solely by name; describe more complex techniques in the Methods section.*
☒	☐	A description of all covariates tested
☐	☒	A description of any assumptions or corrections, such as tests of normality and adjustment for multiple comparisons
☐	☒	A full description of the statistical parameters including central tendency (e.g. means) or other basic estimates (e.g. regression coefficient) AND variation (e.g. standard deviation) or associated estimates of uncertainty (e.g. confidence intervals)
☐	☒	For null hypothesis testing, the test statistic (e.g. *F*, *t*, *r*) with confidence intervals, effect sizes, degrees of freedom and *P* value noted<br>*Give P values as exact values whenever suitable.*
☒	☐	For Bayesian analysis, information on the choice of priors and Markov chain Monte Carlo settings
☒	☐	For hierarchical and complex designs, identification of the appropriate level for tests and full reporting of outcomes
☐	☒	Estimates of effect sizes (e.g. Cohen's *d*, Pearson's *r*), indicating how they were calculated

Our web collection on statistics for biologists contains articles on many of the points above.

Software and code

Policy information about availability of computer code

Data collection	Zen 3.4 Blue Edition for image capture and quantification of fluorescence intensity.<br>Western blot and dot blots were captured using ImageStudio software version 5.2.
Data analysis	4T1 micronuclei and RPE-1 ATAC-Seq data were analyzed using the tools below<br>samtools v1.9<br>bedtools v2.25.0-24<br>macs2 v2.1.2<br>picard v2.8.0<br>deeptools v3.4.2<br>Packages used:<br>DESeq2 1.32.0<br>ChIPSeeker 1.28.3<br>pheatmap 1.0.12<br>ggplot2 3.3.5<br>GenomicRanges 1.44.0<br>clusterProfiler 4.0.5<br>TxDb.Hsapiens.UCSC.hg38.knownGene 3.13.0<br>TxDb.Mmusculus.UCSC.mm10.knownGene 3.10.0<br>org.Hs.eg.db 3.13.0<br><br>Micronuclei WGS data were analyzed using:<br>HMMCopy v1.24.0,<br>MoCaSeq, v0.4.53<br>NovoSort MarkDuplicates (v3.08.02)

bedtools (version 2.25.0)

DLD-1 CUT&RUN, ATAC-Seq and WGS was analyzed using the tools below:
Samtools (version 1.13), 'removeChrom' script from https://github.com/harvardinformatics/ATAC-seq,
Python (v 3.9.4) with packages: Pandas (v 1.3.3), Numpy (v 1.20.1), scipy (1.7.0), CNVKit (0.9.7). bowtie2 v2.4.4
AmpliconSuite (https://github.com/jluebeck/AmpliconSuite-pipeline)

RPE-1 long-term WGS was analyzed using Basepair CNV GATK4 pipeline (https://www.basepairtech.com/).
CUT&RUN data were analyzed using Basepair pipeline: (BETA) EpiCypher - CUT&RUN Peaks, Motif (MACS2, Homer) (https://www.basepairtech.com/).
RNA-Seq FPKM values were obtained from the STAR pipeline in Basepair (https://www.basepairtech.com/).

PCAs for RNA-Seq data were calculated and generated using Partek Flow software version 10.0.
Geneset enrichment analysis were done using GSEA software v4.2.3.
FLIM data was analyzed using SimFCS-64 software, version 03-10-2020.
Histone mass spectrometry data were analyzed using EpiProfile 2.0.
Dot blot quantification was done on ImageStudio software version 5.2.
Flow cytometry analysis was done on FlowJo software version 10.

For manuscripts utilizing custom algorithms or software that are central to the research but not yet described in published literature, software must be made available to editors and reviewers. We strongly encourage code deposition in a community repository (e.g. GitHub). See the Nature Portfolio guidelines for submitting code & software for further information.

Data

Policy information about availability of data

All manuscripts must include a data availability statement. This statement should provide the following information, where applicable:

- Accession codes, unique identifiers, or web links for publicly available datasets
- A description of any restrictions on data availability
- For clinical datasets or third party data, please ensure that the statement adheres to our policy

The ATAC-Seq, CUT and RUN and WGS generated in this study is publicly available (GEO accession code accession code GSE186589, SRA accession code PRJNA882761,). Mass spectrometry raw files are deposited in the repository Chorus (https://chorusproject.org/) under the project number 1790. WGS files from EGA (dataset ID EGAD0000100416) was used in DLD-1 CEN-SELECT samples analysis. The TCGA dataset used was Breast Invasive Carcinoma (TCGA, PanCancer Atlas, https://www.cbioportal.org/study/summary?id=brca_tcga_pan_can_atlas_2018).

Field-specific reporting

Please select the one below that is the best fit for your research. If you are not sure, read the appropriate sections before making your selection.

☒ Life sciences ☐ Behavioural & social sciences ☐ Ecological, evolutionary & environmental sciences

For a reference copy of the document with all sections, see nature.com/documents/nr-reporting-summary-flat.pdf

Life sciences study design

All studies must disclose on these points even when the disclosure is negative.

Sample size	No statistical methods were used to pre-determine sample size. Sample sizes were determined based on prior experience and were chosen as more than or equal to three biological or technical replicates. For micronuclei ATAC-Seq and CUT&RUN, biological duplicates were performed.
Data exclusions	In FLIM experiment where MATLAB function “isooutlier” was used to clear outliers. Outliers (defined as elements more than 1.5 interquartile ranges above the upper quartile or below the lower quartile) were excluded to remove inconsistent lifetime values caused by poor signal-to-noise ratio. There was no exclusion in all other data.
Replication	Experimental and biological replicates are mentioned in figure legends and methods section.
Randomization	Not applicable to this study as no intervention was performed, therefore, it is not possible to randomize.
Blinding	Investigator was not blinded as difference was clear from staining as well as morphology for micronuclei counting and chromosomal missegregation scoring. Therefore blinding was not possible in the experiments.

Reporting for specific materials, systems and methods

We require information from authors about some types of materials, experimental systems and methods used in many studies. Here, indicate whether each material, system or method listed is relevant to your study. If you are not sure if a list item applies to your research, read the appropriate section before selecting a response.

Materials & experimental systems

n/a	Involved in the study
☐	☒ Antibodies
☐	☒ Eukaryotic cell lines
☒	☐ Palaeontology and archaeology
☒	☐ Animals and other organisms
☐	☒ Human research participants
☒	☐ Clinical data
☒	☐ Dual use research of concern

Methods

n/a	Involved in the study
☒	☐ ChIP-seq
☐	☒ Flow cytometry
☒	☐ MRI-based neuroimaging

Antibodies

Antibodies used

Primary Antibodies: H3K4Me3 (Abcam, ab8580, Lot#GR3362386-1, 1:1000), H3K9Me3 (Abcam, ab8898, Lot#GR3217595-2, 1:500), H3K14Ac (Abcam, 52946, Lot#GR3252548-3, 1:250), H3K27Me3 (Active Motif, 61017, lOT#15819018, 1:1000), H3K27Ac (Abcam, ab4729, Lot# GR3374555-1, 1:1000), H3K36Me2 (Cell Signaling Technology, C75H12, Lot#5, 1:200), H3K36Me3 (Abcam, ab9050, Lot#GR3307136-1, 1:1000), H2AK119Ub (Cell Signaling Technology, 8240, Lot#8, 1:1600), H2BK120Ub (Cell Signaling Technology, 5546S, Lot#7, 1:1600), human cGAS (LSBio, LS-C757990, Lot#164559, 1:1000), mouse cGAS (Cell Signaling Technology, 31659S, Lot#5, 1:1000), Lamin B2 (Abcam, ab151735, Lot#GR108766-17, 1:2000), Actin (Abcam, ab6276, Lot#GR3324554, 1:10000), H2A (Invitrogen, MA3-047, Lot#RD230767, 1:1000), H2B (Abcam, ab193203, Lot#GR3232535-11, 1:1000), KDM1A (Abcam ab17721, Lot#GR3440282-1, 1:1000), PHC2 (Thermo Fisher Scientific, PA5-61504, Lot#PA5-61504, 1:50), RNF40 (Abcam ab126959, Lot#GR111878-8, 1:250), pRpb1 CTD(ser5) (Cell Signaling Technology, 13523S, Lot#1, 1:250), Lamin A Thermo (Fisher Scientific MA1-06101, Lot#XB3509871, 1:1000).

Secondary antibodies: Alexa fluor plus 594 donkey anti mouse (Invitrogen, A32744, Lot#XC343358, 1:1000), Alexa fluor plus 594 donkey anti rabbit (Invitrogen, A32754, Lot#UI290268, 1:1000), Alexa Fluor plus 488 donkey anti mouse (Invitrogen, A32766, Lot#VE298230, 1:1000), Alexa Fluor plus 488 donkey anti rabbit (Invitrogen, A32790, Lot#XJ357262, 1:1000), IRDye 680 RD goat anti mouse (LI-COR, 926-68070, Lot# D01209-05, 1:1000), IRDye 680 CW donkey anti rabbit (LI-COR, 926-32213, Lot#D20920-35, 1:1000), Biotinylated anti- mouse secondary (Vector Labs, MKB-2225-10-1, lot#ZF-0122, 5.75ug/mL), Biotinylated goat anti-rabbit IgG (Vector labs, cat# 30014, lot#ZG0312, 5.75ug/mL).

Validation

The primary antibodies used were purchased from reputable sources validated for the species and application (immunoblotting and/or immunofluorescence) by their respective manufacturers in their website's validation statements. The validations were done using recombinant protein and/or cell lines known to express the target protein as a positive control. Moreover antibodies for H3K27me3, H3K14ac, H3K27ac was futher validated using pharmacological inhibition in this manuscript as show in figures 1c and extended data figure 2b.

Eukaryotic cell lines

Policy information about cell lines

Cell line source(s)

MCF10A (CRL-10317), MDA-MB-231 (HTB-26), 4T1 (CRL-2539), RPE-1 (CRL-4000), and HEK-293T (CRL-3216) cells were purchased from American Type Culture Collection (ATCC). RPE-1 p53KO, and 4T1 TREX1KO were kind gifts from the Maciejowski lab at MSKCC and were purchased from ATCC prior. OVCAR-3 (HTB-16) was a kind gift from Jose Dopeso Gonzales and were purchased from ATCC prior.

Authentication

All cell lines used in this manuscript were authenticated by ATCC which used morphology, karyotyping and PCR-based techniques.

Mycoplasma contamination

All cell lines tested negative for mycoplasma according to the test kit from R&D Systems™ (MycoProbe Mycoplasma Detection Kit).

Commonly misidentified lines
(See ICLAC register)

None.

Human research participants

Policy information about studies involving human research participants

Population characteristics

The study cohort (MSK SPECTRUM) includes 42 women with newly diagnosed, treatment-naive high-grade serous ovarian cancer (HGSOC). Patients between the ages of 39 and 81 at diagnosis (median age: 61 years). 6 out of 42 cases had BRCA1 mutations (14%) and 1 out of 42 cases had a BRCA2 mutation (2%).

Recruitment

All enrolled patients were consented to an institutional biospecimen banking protocol and a protocol to perform targetted panel sequencing (MSK-IMPACT). All analyses were performed per a biospecimen research protocol. All protocols were approved by the Institutional Review Board (IRB) of Memorial Sloan Kettering Cancer Center (MSKCC). Patients were consented following the IRB-approved standard operating procedures for informed consent. Written informed consent was obtained from all patients before conducting any study-related procedures. This study was conducted in accordance with the Declaration of Helsinki and the Good Clinical Practice guidelines (GCP).

Ethics oversight	Institutional Review Board (IRB) at Memorial Sloan Kettering Cancer Center (MSKCC).

Note that full information on the approval of the study protocol must also be provided in the manuscript.

Flow Cytometry

Plots

Confirm that:

☒ The axis labels state the marker and fluorochrome used (e.g. CD4-FITC).

☒ The axis scales are clearly visible. Include numbers along axes only for bottom left plot of group (a 'group' is an analysis of identical markers).

☒ All plots are contour plots with outliers or pseudocolor plots.

☒ A numerical value for number of cells or percentage (with statistics) is provided.

Methodology

Sample preparation	Mentioned in the method section under "micronuclei purification". Briefly, cells were expanded in 245 × 245 × 25 mm dishes and treated with reversine as previously specified. Cells were then harvested and washed DMEM. Washed cells were resuspended in pre-warmed (37 °C) DMEM supplemented with 10 µg/ml cytochalasin B (Sigma-Aldrich) at a concentration of around 5x106 cells/mL DMEM and incubated at 37°C for at least 30 minutes. Subsequently, cells were centrifuged at 300g for 5 minutes and resuspended in cold lysis buffer (10 mM Tris-HCl, 2 mM Mg-acetate, 3 mM CaCl2, 0.32 M sucrose, 0.1 mM EDTA, 0.1 % (v/v) NP-40, pH 8.5) freshly complemented (with 1 mM dithiothreitol, 0.15 mM spermine, 0.75 mM spermidine, 10 µg/ml cytochalasin B and protease inhibitors) at a concentration of 107 cells/ml lysis buffer. Resuspended cells were then dounce-homogenized 10 times using a loose-fitting pestle. Then, lysates were mixed with an equal volume of cold 1.8 M sucrose buffer (10 mM Tris-HCl, 1.8 M sucrose, 5 mM Mg-acetate, 0.1 mM EDTA, pH 8.0) freshly complemented (with 1 mM dithiothreitol, 0.3 % BSA, 0.15 mM spermine, 0.75 mM spermidine) before use. In a 50 mL conical tube, 10 mL of the mixture was then layered on top of a two-layer sucrose gradient (20 mL of 1.8 M sucrose buffer on top of 15 mL 1.6 M sucrose buffer). This mixture was then centrifuged in a swing bucket centrifuge at 950g for 20 min at 4°C. The first resulting 2 mL top fraction is discarded; next 5–6 mL mostly contain micronuclei is collected, the next 3 mL mostly containing primary nuclei is also collected in a separate container. Collected fractions were diluted 1:5 with FACS buffer (ice cold PBS freshly supplemented with 0.3 % BSA, 0.1 % NP-40 and protease inhibitors). Diluted MN were then centrifuged at 950g in JS-5.2 swing bucket centrifuge for 20 min at 4 °C. The resulting supernatant was removed by aspiration and either micronuclei or primary nuclei was resuspended in 2–4 mL of FACS buffer supplemented with 2 µg/ml DAPI (however, no DAPI was used for micronuclei purification for ATAC-seq experiments). Resuspended samples were filtered through a 40 µm ministrainer (PluriSelect) into FACS tubes.
Instrument	FACSAria III Cell Sorter
Software	FlowJo
Cell population abundance	N/A (Nuclei was sorted instead of cells)
Gating strategy	Mentioned in the method section under "micronuclei purification". Micronuclei were sorted by FACSAria (BD Biosciences) into FACS buffer at the MSKCC Flow Cytometry Core Facility. Default FSC and DAPI thresholds were lowered, and a log scale was used to visualize MN population. Population in the area that has lower FSC and DAPI signals is gated for micronuclei while higher FSC and DAPI signals is gated for primary nuclei. From micronuclei gate, the population in the area that has mCherry positive, low GFP signal is gated for intact micronuclei, while the higher GFP signal is gated for ruptured micronuclei. The figure exemplifying the gating strategy is provided in extended data figure 6e.

☒ Tick this box to confirm that a figure exemplifying the gating strategy is provided in the Supplementary Information.
